# Targeted and untargeted quantification of quorum sensing signalling molecules in bacterial cultures and biological samples via HPLC-TQ MS techniques

**DOI:** 10.1007/s00216-020-03040-6

**Published:** 2020-11-18

**Authors:** Federica Dal Bello, Michael Zorzi, Riccardo Aigotti, Davide Medica, Vito Fanelli, Vincenzo Cantaluppi, Eleonora Amante, Viviana Teresa Orlandi, Claudio Medana

**Affiliations:** 1 Department of Molecular Biotechnology and Health Sciences, University of Turin, via Pietro Giuria 5, 10, 125 Torino, Italy; 2 Department of Surgical Science Anesthesia and Critical Care, AOU Città della Salute e della Scienza, University of Turin, Corso Bramante 88, 10, 126 Torino, Italy; 3 Department of Translational Medicine, University of Eastern Piedmont, Via Solaroli, 17, 28, 100 Novara, Italy; 4 Department of Chemistry, University of Turin, via Pietro Giuria 5, 10, 125 Torino, Italy; 5 Department of Biotechnology and Life Sciences, University of Insubria, Via J.H. Dunant 3, 21, 100 Varese, Italy

**Keywords:** Quorum sensing molecules, Homoserine lactones, Hydroxyquinolones, *Pseudomonas aeruginosa*, Mass spectrometry, Triple quadrupole

## Abstract

Quorum sensing (QS) is the ability of some bacteria to detect and to respond to population density through signalling molecules. QS molecules are involved in motility and cell aggregation mechanisms in diseases such as sepsis. Few biomarkers are currently available to diagnose sepsis, especially in high-risk conditions. The aim of this study was the development of new analytical methods based on liquid chromatography-mass spectrometry for the detection and quantification of QS signalling molecules, including N-acyl homoserine lactones (AHL) and hydroxyquinolones (HQ), in biofluids. Biological samples used in the study were *Pseudomonas aeruginosa* bacterial cultures and plasma from patients with sepsis. We developed two MS analytical methods, based on neutral loss (NL) and product ion (PI) experiments, to identify and characterize unknown AHL and HQ molecules. We then established a multiple-reaction-monitoring (MRM) method to quantify specific QS compounds. We validated the HPLC-MS-based approaches (MRM-NL-PI), and data were in accord with the validation guidelines. With the NL and PI MS-based methods, we identified and characterized 3 and 13 unknown AHL and HQ compounds, respectively, in biological samples. One of the newly found AHL molecules was C12-AHL, first quantified in *Pseudomonas aeruginosa* bacterial cultures. The MRM quantitation of analytes in plasma from patients with sepsis confirmed the analytical ability of MRM for the quantification of virulence factors during sepsis.

**Supplementary Information:**

The online version contains supplementary material available at 10.1007/s00216-020-03040-6.

**Figure:**
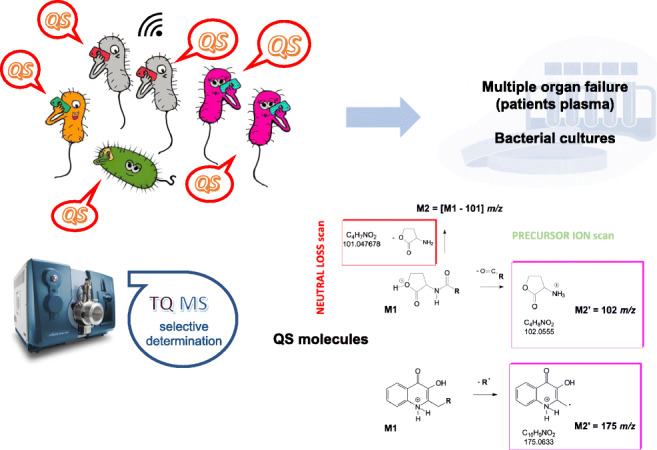
Graphical abstract

## Introduction

Bacteria have the ability to interact with each other through a complex language called “quorum sensing” (QS) [1–3]. Literally, QS means “detection of the quorum”, and it refers to the ability of bacteria to monitor their population density, and consequently to control their gene expression, through the control of the amount of specific molecules, called autoinducers (AIs), in their living environment [4–6]. AIs are small and diffusible molecules produced by bacteria, released and accumulated in the extracellular environment. When many AIs are produced and stored, and their concentration reaches a threshold level (the quorum), the bacterial population is able to activate or to repress target genes [7]. This mechanism enables the survival of a bacterial population in a constantly changing environment (temperature, pH and osmotic concentration variations, and nutrient availability) thanks to the synthesis of new proteins. QS-mediated changes are energetically expensive, and they are advantageous only when cells have reached a high-density population [8, 9].

**Fig. 1:**
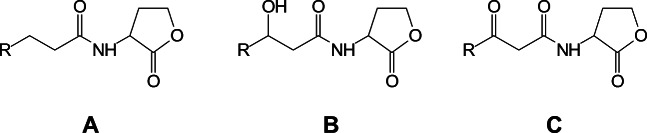
N-Acyl homoserine lactone generic structure. **a** Non-substituted N-acyl homoserine-L-lactone (Cn-HSL) acyl chain; **b** N-(3-hydroxyacylhomoserine)-L-lactone (3-OH-Cn-HSL) acyl chain; **c** N-(3-oxoacylhomoserine)-L-lactone (3-oxo-Cn-HSL) acyl chain. The length is variable, generally *n* = 4–14

Gram-positive and Gram-negative bacteria use different means of communication [10]. Gram-positive bacteria produce oligopeptides as QS autoinducer molecules, while Gram-negative bacteria use others QS signal molecules [11–13]. The most abundant and common are N-acyl homoserine lactones (AHLs) [14, 15]. These molecules are characterized by a γ-lactone cycle which is N-acylated in the α position and an acyl chain (indicated as R-chain in Fig. 1). Chain length is the signal specificity factor for bacteria, and the chain typically contains between 4 and 16 carbon atoms. The presence of an oxo or a hydroxy group linked to the third carbon atom of the chain is a further element of distinction.

In some human opportunistic pathogens, like *Pseudomonas aeruginosa*, the secretion of the most abundant AHL QS signalling molecules N-(3-oxododecanoyl)-L-homoserine lactone (3-oxo-C12-AHL) and N-butanoyl-L-homoserine lactone (C4-AHL) depends on the regulatory circuit systems *Las* or *Rhl* [16, 17]. In bacteria, those systems control the expression of different virulence genes in a population density-dependent manner.

**Fig. 2:**
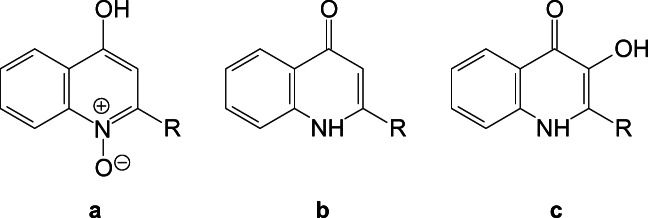
Generic structure of quinolone signalling molecules. **a** 2-Alkyl-4-hydroxyquinolone N-oxide with a variable chain length (C7–C9); **b** 2-alkyl-4(1H)-quinolone alkyl chain (the chain length is variable, C7–C11); **c** 2-alkyl-3-hydroxy-4(1H)-quinolone

In addition to AHLs, Gram-negative bacteria such as *Pseudomonas aeruginosa* use a hydroxyquinolone molecule (HQ), the 2-heptyl-3-hydroxy-4(1H)-quinolone (known as PQS or C7 HQ), as a QS signalling compound [18, 19]. The basic structure of quinolone molecules consists of a bicyclic ring. The length of the acyl chain (marked as R-chain in Fig. 2) ranges between seven and 11 carbon atoms; the presence of a hydroxy or a carbonyl group in position 1 of the bicyclic ring is a further element of distinction.

As described above, AHLs and HQs are the most abundant QS signalling molecules produced by Gram-negative bacteria. Many studies in the literature have aimed to describe and quantify well-known or new QS molecules in bacterial culture or human samples [20–35]. There are two main methods for obtaining the measurements: biosensors [21–23, 26, 30, 31] and methods based on liquid chromatography coupled to (high-resolution) mass spectrometry [20, 23–25, 27–29, 32–36]. Biological biosensors reach very low sensitivity, with a pg/mL (fmol) limit of detection, and are highly specific for a single QS (AHL or HQ). However, they miss unknown molecules, and the quantitation demonstrates low accuracy [32]. On the contrary, mass spectrometry-based methods, both targeted and untargeted, offer accurate quantitation of AHL and HQ compounds. During the past few years, many LC-MS methods have been developed that exploit several stationary and mobile phases, using both targeted and untargeted MS approaches. Some studies showed a very low limit of quantitation (pg/mL) and good selectivity, optimizing a short liquid chromatography separation both for AHLs, furthermore enantiomeric, and for HQs [27–29, 33–36]. Other research groups have developed extended chromatography separation runs to provide the quantitation of a higher number of molecules [24] or to identify new AI molecules with an untargeted high-resolution MS approach [32].

Summarizing, there are a huge number of QS molecules produced by bacteria, and the identification, characterization and accurate quantitation of peculiar and unknown QS are still needed.

The purpose of this work was to develop and validate HPLC-TQ MS chemical class-specific methods able to identify and quantify quorum sensing molecules (AHLs and HQs) in different matrices, such as bacterial cultures and biological plasma samples. In order to characterize and identify unknown AHL and HQ signalling molecules, the aim of the research was the development of an MS analytical method based on neutral loss (NL) and product ion (PI) experiments. A tandem mass spectrometry method based on a multiple reaction monitoring (MRM) approach was developed for the quantitation of AHLs and HQs in bacterial cultures and biological plasma samples. To ensure greater reliability of analytical data, fragmentation pathways of analytes of interest and the exact mass of detected unknown molecules were confirmed by high-resolution mass spectrometry (LTQ-Orbitrap). The developed MRM method was firstly applied to quantify the QS molecules in *Pseudomonas aeruginosa* cultures obtained from two different strains [37], and with our NL and PI methods we aimed to elucidate whether other QS molecules were involved in the protection mechanism used by bacteria. Secondly, the MRM method was applied to pathology plasma samples (before and after hemoperfusion) of patients affected by sepsis-related multi-organ failure (MOF), which is associated with high mortality. To our knowledge, this study is the first to provide a tandem mass spectrometry quantitation of both AHLs and HQs in plasma samples from patients with sepsis.

## Materials and methods

### Chemicals

Analytical standards (purity >98%) of 2-heptyl-3-hydroxy-4(1H)-quinolone (C7 HQ), N-(3-oxododecanoyl)-L-homoserine lactone (3-oxo-C12-AHL), N-hexanoyl-L-homoserine lactone-D3 (ND3) and N-butanoyl-L-homoserine lactone (C4-AHL) were purchased from Merck KGaA (Darmstadt, Germany). Stock solutions were prepared with a concentration of 1000 mg/L using methanol and were stored at −4 °C until use. Further dilutions were obtained in 0.1% formic acid in water/acetonitrile 60:40. All aqueous solutions were prepared with HPLC-grade water from a Milli-Q Academic System (Millipore, Burlington, MA, USA). Ethyl acetate HPLC-MS grade, acetonitrile and methanol hypergrade for LC-MS, and formic acid were purchased from VWR International (Radnor, PA, USA).

### Instrumentation

Separation and analysis of all analytes and samples were achieved upon an HPLC-TQ MS platform. HPLC consisted of a Shimadzu Nexera X2 Ultra High Performance Liquid Chromatography (UHPLC) system (Shimadzu, Kyoto, Japan) coupled for identification and quantitation to a QTRAP 5500 system (Sciex, Darmstadt, Germany). The triple quadrupole was equipped with a Turbo V™ Source (ESI mode) which utilized nitrogen and air as sheath and reagent gas, respectively. To verify the exact mass values, we used an LTQ-Orbitrap high-resolution mass spectrometer (Thermo Scientific, Waltham, MA. USA) within mass accuracy of ±3 ppm and resolution of 30 k.

### HPLC parameters

To investigate AHL and HQ signalling molecules in biological samples, different chromatographic gradients were carried out. However, HPLC methods had several features in common. The analytical column used was a Phenomenex Luna C18 reversed-phase column (150 × 2.1 mm i.d., 3 μm particles). Sample injection volume was 10 μL. Autosampler and oven temperatures were set at 15 °C and 45 °C, respectively, for the duration of all analyses.

For AHLs, HPLC-TQ MS analysis eluents were formic acid 0.1% in water (solvent A) and in acetonitrile (solvent B). Flow rate was set at 200 μL/min. For bacterial cultures, the mixture percentage changed from 40% solvent B to 100% solvent B during the first 35 min, maintained for 10 min, and then the column returned to the starting conditions. For plasma samples, the HPLC run started from 40% solvent B, increased to 100% solvent B in 19 min, maintained for 10 min, and then returned to the starting conditions. Bacterial cultures were more complex than plasma samples and required us to obtain a satisfactory chromatographic separation with slower solvent variation during gradient elution (see Electronic Supplementary Material [ESM] Fig. S1).

Chromatographic separation for the MRM and PI analysis of C7 HQ in bacterial cultures and plasma samples was achieved using 2-picolinic acid 2 mM/formic acid 0.1% in water (solvent C) and acetonitrile 0.1% formic acid (solvent D). 2-Picolinic acid acted as a bidentate chelator, preventing peak distortion caused by C7 HQ, an iron chelator molecule [33]. Gradient started from 20% solvent D, up to 100% solvent D in 12 min; then the column returned to the starting conditions. Flow rate was 250 μL/min.

### MS settings

The LC effluent was delivered to the Turbo V™ Source (ESI positive ionization mode) using nitrogen as the sheath (GS1) and curtain (CUR) gas and air as reagent (GS2) gas, respectively. The mass spectrometer parameters were as follow: CUR 26 arbitrary units (arb), GS1 45 arb, GS2 50 arb, ion spray voltage 5.5 kV and ion spray temperature 500 °C.

Considering all the possible MS experiments developed, each sample was analysed five times: MRM-NL-PI for AHL analysis, and MRM-PI for HQ analysis. The MRM, NL and PI parameters are listed in Table 1. For MRM acquisition, we selected one qualitative and one other quantitative (bold in Table 1) transition for each analyte. The instrument parameters are listed in ESM Tables S1 and S2.

**Table 1:** Multiple reaction monitoring (MRM), neutral loss (NL) and product ion (PI) scan parameters for AHL and HQ analysis (bolded transitions were used as quantitative ones)

	Analyte	Precursor ion [M + H]^+^	Product ion [M + H]^+^	Molecule family	MS mode	Δm (Da)
MRM	3-oxo-C12-AHL	**298.2**	**102.2**	AHL	*NL*	101.0
		298.2	197.2		*PI*	102.0
	C4-AHL	**172.1**	**102.2**	HQ	*PI*	175.0
		172.1	71.1			
	C7 HQ	**260.0**	**188.0**			
		260.0	147.0			
	ND3	**203.2**	**102.1**			
		203.2	74.1			

### Bacterial cultures and biological samples

Two *Pseudomonas aeruginosa* strains were selected as representatives of bacterial cultures: wild-type PAO1 strain and its isogenic mutant RhlI defective in the synthesis of C4-AHL previously obtained [37, 38].

Different *Pseudomonas aeruginosa* PAO1 and RhlI bacterial cultures were prepared and analysed: (i) wild-type bacterial culture grown in Luria-Bertani (LB) broth (rich in nutrients), WT-LB; (ii) wild-type bacterial culture grown in the mineral medium (M9) culture (low in nutrients), WT-M9; (iii) RhlI mutant bacterial culture grown in the LB broth, RhlI-LB; and IV) RhlI mutant bacterial culture grown in the M9 culture, RhlI-M9.

For the application of the MRM-NL-PI methods developed, we analysed plasma samples from healthy people and patients affected by MOF. All the patients involved in the present study expressed their consent and their will based on their awareness of the proposed study of their biological fluids, freely deciding whether to accept. All the procedures followed in the work were carried out in accordance with the ethical standards of our institutional, the national research committee and with the Code of Ethics of the World Medical Association (1964 Helsinki declaration).

### Sample preparation and enrichment

All the bacteria media were prepared as described by Orlandi et al. [37]. Bacterial cultures were centrifuged for 10 min at 12,000 rpm and RT, and the supernatants were then collected and filtered (Minisart RC15 Ø 0.20 mm; Sartorius) before extraction (see below).

Samples from patients with MOF were taken during a weekly dialysis session, and they were collected before (t_0_), after 2 h (t_2h_) and after 24 h (t_24h_) of hemoperfusion, and those from healthy people during a draw in the morning. Plasma was separated from blood with an ultracentrifuge and then stored refrigerated at 4 °C until use. Samples were processed and analysed within 2 days.

Before extraction of the analytes from samples, 200 μL of bacterial culture medium or plasma was spiked with ND3 internal standard (IS) to a final concentration of 200 μg/L. The internal standard was used to evaluate injection only. All of the analytical validation was done on the basis of external standard calibration. Samples were extracted twice in succession with 1 mL of ethyl acetate [39]. After each addition of organic solvent, the sample was centrifuged at 5000 × *g* for 5 min at RT, and the organic fractions were collected and dried under a gentle stream of N_2_ with heating at 40 °C. Finally, the residue was reconstituted in 100 μL of 0.1% formic acid in water/acetonitrile 60:40.

### HPLC-TQ MS method validation

The validation procedure was performed on the HPLC-TQ MS platform, according to the European Medicines Agency (EMA) and Eurachem guidelines [40, 41]. The calibration curves were run using a matrix free of quorum sensing molecules (plasma samples from healthy people) by performing the standard addition method. The absence of analytes of interest within the matrix used (QS-free matrix) for the method validation was verified through the LTQ-Orbitrap high-resolution power platform.

For method validation, different parameters were evaluated: selectivity, recovery, carry-over, intra-run accuracy and precision, limit of quantitation (LOQ), lower LOQ (LLOQ), upper LOQ (ULOQ), stability to freeze-thaw cycle and calibration model. The last one, in particular, was evaluated using a stepwise approach as described elsewhere [42], and the linearity of the calibration curves using an R routine developed by Desharnais et al. [43, 44]: Firstly, the heteroscedasticity of data points was tested by performing an *F*-test on the variance of the area ratios at the lowest and highest calibration levels. The heteroscedasticity study was also integrated with the Levene test (in the version modified by Brown and Forsythe). Then, a partial *F*-test was used to evaluate whether the calibration model followed a linear or quadratic trend [45]. The goodness of fit of the calibration model was finally evaluated by studying the normality of the standardized residuals (with the Cramer von Mises test) and by performing the back-calculation on the averaged signal from four replicates [43, 44].

For the validation of AHL molecules, C4-AHL and 3-oxo-C12-AHL were selected as molecule class representatives, whereas C7 HQ was used as a representative of HQ molecules. For all the analyses, ND3 was used as injection standard.

Selectivity (SEL%) was evaluated by comparing the chromatograms of six individual QS-free matrices and could be below 20%. Recovery (REC%) was evaluated by relating the responses of analytes in the extracted samples to those solubilized in the injection solvent. The experiments were conducted at LLOQ and ULOQ concentrations, depending on the analyte. REC% was evaluated only for the MRM approach. The carry-over effect (CO%) was studied by comparing the signal of the molecule of interest in QS-free matrix after the injection of the highest concentration point of the calibration curve. Values could be below 20% of the LLOQ value. Accuracy (BIAS%) and precision (variation coefficient CV%) of intraday (repeatability) were calculated in QS-free matrix samples at three different concentrations carried out five times. Values could be below 15%. Limit of quantitation (LOQ) was determined by ten times the signal-to-noise ratio, expressed as the absolute value of analyte concentration. LLOQ and ULOQ were expressed as the experimentally lower and upper measured analyte concentrations, respectively. Freeze-thaw stability (STAB%) was evaluated by comparing freshly prepared processed samples frozen at −20 °C for three cycles and thawed at RT in order to determine the freeze-thaw stability of the AHL and HQ standards. The experiments were evaluated at two concentrations, LLOQ and ULOQ. STAB% ranged between 85% and 115%.

## Results

### Validation results

Validation results are summarized in Table 2 and presented in ESM Section 2, “Validation tables and results”. All parameters conformed to those suggested by EMA and Eurachem guidelines.

**Table 2:** Validation parameters for 3-oxo-C12-AHL, C4-AHL and C7 HQ obtained in MRM, NL, PI HPLC-MS approaches (NM = not measured)

Validation parameter	Conc. (μg/L)	3-oxo-C12-AHL			C4-AHL			C7 HQ	
		MRM	NL	PI	MRM	NL	PI	MRM	PI
SEL%		8.12	0.50	1.10	5.65	1.63	2.54	16.5	4.73
REC%	LLOQ	45.5	NM	NM	51.0	NM	NM	49.9	NM
	ULOQ	53.7	NM	NM	60.9	NM	NM	34.3	NM
CO%		7.10	0.57	1.29	8.90	1.65	0.87	16.1	2.98
I-R BIAS%	LLOQ	1.65	3.66	2.40	0.61	2.72	3.59	2.11	2.28
	50.0	9.88	7.77	3.05	5.32	1.84	4.32	2.27	8.68
	ULOQ	1.61	3.63	4.97	0.23	0.80	6.98	4.14	2.27
I-R CV%	LLOQ	12.4	0.87	12.9	18.4	25.0	13.7	26.5	11.9
	50.0	17.1	8.87	5.91	10.8	19.1	16.6	18.4	1.72
	ULOQ	14.3	4.98	12.0	15.6	20.9	18.1	20.7	5.84
LOQ	μg/L	0.090	0.293	0.117	0.271	0.457	0.066	0.151	0.385
LLOQ	μg/L	0.40	5.00	1.00	0.40	5.00	1.00	0.40	1.00
ULOQ	μg/L	400	400	300	400	400	300	200	300
STAB%	LLOQ	106	89.5	86.0	97.3	91.1	88.9	115	90.2
	ULOQ	101	88.6	90.2	102	86.9	95.5	117	96.2

Selectivity of each MS approach was satisfactory with a value always below 20%, ranging between 0.50 and 16.5%. No isomeric or isobaric interfering compound co-eluted with the analytes, and no ion suppression was observed for the AHL and HQ standard molecules.

The recovery calculated for the MRM approach ranged between 34% and 61%. This was presumably correlated with the presence of esterase enzymes in the plasma matrices, and quorum sensing molecules, being esters, underwent partial hydrolysis. However, using the calibration curves obtained in plasma matrices, we overcame the issue.

Carry-over effect measurements demonstrated, for all MS methods, that in a QS-free matrix sample, the area under the curve (AUC) of the analytical standards was lower than 20% of the corresponding LLOQ area.

The precision and accuracy of intraday runs were below 20% for the selected curve calibration points.

LOQ values for analytes ranged between 0.09 and 0.457 μg/L, and the MRM method showed the best LLOQ value (0.4 μg/L).

Stability test parameters always fell within the acceptable limits, and based on the results, the average stability % value at each concentration level ranged between 85% and 117%. Therefore, the plasma matrix was similarly stable at operative condition temperatures as in freezing and thawing cycles without affecting the concentration of the analyte.

The study of the calibration model provided concordant results for all the analytes. Concerning the study of heteroscedasticity, the *F*-test and the Levene test provided consistent results for all but two calibration sets, namely C4-AHL-NL and C4-AHL-PI, for which the Levene test suggested a homoscedastic trend (ESM Table S5). In both cases, a more conservative approach was preferred, and the weight was applied as suggested by the routine from Desharnais. The weight for the heteroscedasticity was equal to 1/x^2^ in all cases. Furthermore, all the calibration curves were confirmed to be linear, with *p* values for the partial *F*-test above the significance limit of 0.05. The goodness of the calibration models was proved by the good results provided by the Cramer von Mises test (*p* values always not significant) and the back-calculation (deviations far below ±25% for all the models). Finally, also the R-squared values *(*R^2^ > 0.990) demonstrated how our model explained all of the variation in the response variable around its mean.

### Sample results

With the development of three different MS approaches, we detected and quantified both unknown and known AHL and HQ molecules in biological samples.

**Fig. 3:**
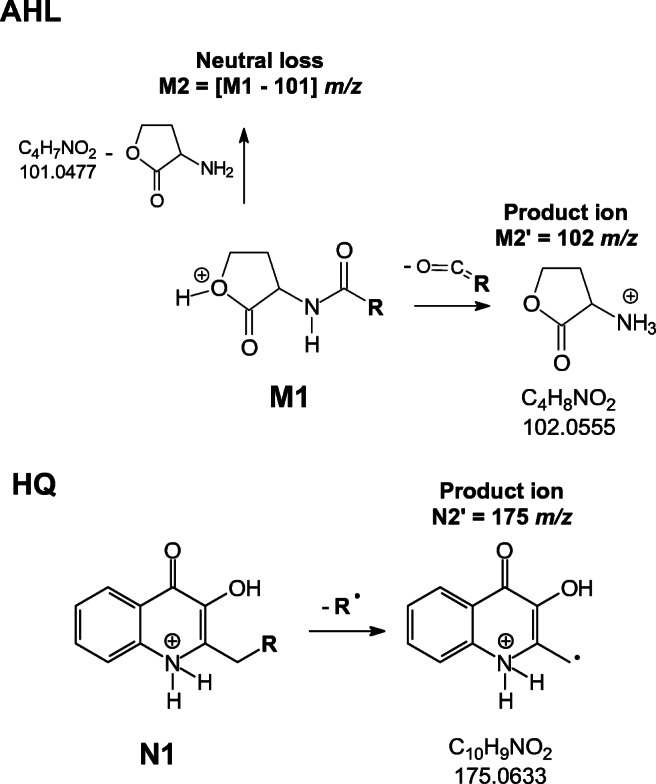
TQ MS/MS selective acquisition modes. M1 is a generic structural formula of a protonated molecule belonging to the N-acyl homoserine lactones (AHLs) family. R varies between 4 and 16 carbon atoms. M2 is the product ion formed after the neutral loss of a 2-amino-gamma butyrolactone molecule (MW 101 Da). M2′ is the product ion with *m/z* 102. Analogously, N1 is a general structural formula of compounds belonging to the hydroxyquinolone signalling molecule (HQ) family. R varies between 7 and 11 carbon atoms. N2′ is the product ion with *m/z* 175

For AHL unknown structures, we used the NL and PI approaches. In NL experiments, precursor and product ions were monitored for the loss of 101 Da which corresponds to 2-amino-gamma butyrolactone. In PI mode, Q3 was set to detect only *m/z* 102 corresponding to the elemental composition of protonated 2-amino-gamma butyrolactone (Fig. 3).

For new molecules belonging to the HQ family, we used a PI methodology selecting as product ion the protonated radical ion 2-methyl-3-hydroxyquinolin-4-one with an *m/z* ratio of 175 (Fig. 3).

Unknown AHL and HQ signalling molecules, identified and characterized in an untargeted approach using NL and PI modes, were confirmed by the use of the HRMS tool (LTQ-Orbitrap), and to better describe them we studied the fragmentation pathways of analytical standards (see “Discussion” section).

Two unknown AHL species were detected both in *P. aeruginosa* bacterial cultures and patients’ plasma samples: 3-oxo-C10-AHL (C_14_H_23_NO_4_, *m/z* 270.1705) and C12-AHL (C_16_H_30_NO_3_, *m/z* 284.2226). One other, C6-AHL (C_10_H_17_NO_3_*, m/z* 200.1287), was present in bacterial cultures only (see ESM for chemical and structural formulas, Table S3).

In the bacterial cultures, 13 unknown HQ molecules were identified by the PI approach and confirmed by HRMS analysis: C2-HQ (C_11_H_11_NO_2_, *m/z* 190.0868), C3-HQ (C_12_H_13_NO_2_, *m/z* 204.1024), C4-HQ (C_13_H_15_NO_2_, *m/z* 218.1181), C5-HQ (C_14_H_17_NO_2_, *m/z* 232.1337), C6-HQ (C_15_H_19_NO_2_, *m/z* 246.1494), C6:1-HQ (C_15_H_17_NO_2_, *m/z* 244.1337), C7:1-HQ (C_16_H_19_NO_2_, *m/z* 258.1494), C8-HQ (C_17_H_23_NO_2_, *m/z* 274.1807), C8:1-HQ (C_17_H_21_NO_2_, *m/z* 272.1650), C9-HQ (C_18_H_25_NO_2_, *m/z* 288.1936), C9:1-HQ (C_18_H_23_NO_2_, *m/z* 286.1807), C11-HQ (C_20_H_29_NO_2_, *m/z* 316.2276) and C11:1-HQ (C_20_H_27_NO_2_, *m/z* 314.2120) (see ESM for chemical and structural formulas, Table S4).

The detected compounds were then semi-quantified in bacterial cultures using the validated calibration curves of C4-AHL and 3-oxo-C12-AHL, depending on chain length and on hydroxylation grade, to quantify AHL molecules, and of C7 HQ to quantify HQ compounds. For the quantification of both AHL and HQ molecules, we used the calibration curves obtained with the PI MS methods because of their lower LLOQ (1 μg/L for PI vs. 5 μg/L for NL). The results are listed in Tables 3 (AHL) and 4 (HQ).

**Table 3:** *Pseudomonas aeruginosa* culture concentrations (expressed in μg/L) of the unknown AHL molecules semi-quantified using the linear equation of the PI validated MS approach

Concentration (μg/L)			
	C6-AHL	3-oxo-C10-AHL	C12-AHL
WT-LB	6.57	0.62	0.13
WT-M9	ND	0.72	0.70
RhlI-LB	ND	0.52	ND
RhlI-M9	ND	2.89	ND

*WT* wild type, *RhlI* mutant bacterium, *M9* mineral medium, *LB* Luria-Bertani broth, *ND* not detectable (<LLOQ).

**Table 4:** *Pseudomonas aeruginosa* culture concentrations (expressed in μg/L) of the unknown HQ molecules semi-quantified using the linear equation of the PI validated MS approach

	Concentration (μg/L)					
	C2 HQ	C3 HQ	C4 HQ	C5 HQ	C6 HQ	C6:1 HQ
WT-LB	ND	0.76	3.96	16.11	38.54	0.33
WT-M9	ND	ND	ND	ND	ND	ND
RhlI-LB	11.15	98.76	33.14	232.45	145.45	17.02
RhlI-M9	ND	ND	ND	ND	ND	ND
	C7:1 HQ	C8 HQ	C8:1 HQ	C9 HQ	C9:1 HQ	C11 HQ
WT-LB	24.07	81.44	1.93	182.57	26.98	1.57
WT-M9	ND	ND	ND	ND	ND	ND
RhlI-LB	2.00	45.75	3.61	12.46	7.40	ND
RhlI-M9	ND	ND	ND	ND	ND	ND
	C11:1 HQ					
WT-LB	28.60					
WT-M9	ND					
RhlI-LB	1.48					
RhlI-M9	ND					

*WT* wild type, *RhlI* mutant bacterium, *M9* mineral medium, *LB* Luria-Bertani broth, *ND* not detectable (<LLOQ)

Finally, the known analytes (C4-AHL, 3-oxo-C12-AHL and C7 HQ) were quantified in bacterial cultures and patients’ plasma samples with the validated HPLC-TQ MRM MS method, and the results are shown in Tables 5 and 6.

**Table 5:** Analyte concentrations (μg/L) measured in bacterial cultures of *Pseudomonas aeruginosa* with the MRM approach

	Concentration (μg/L)		
	C4-AHL	3-oxo-C12-AHL	C7 HQ
WT-LB	5.36	3.66	357
WT-M9	nd	2.07	nd
RhlI-LB	nd	2.17	736
RhlI-M9	nd	1.16	nd

**Table 6:** Analyte concentrations (μg/L) measured in plasma samples of patients with multi-organ failure (MOF) pathology with the MRM approach

Concentration (μg/L)						
	MOF#1			MOF#2		
	C4-AHL	3-oxo-C12-AHL	C7 HQ	C4-AHL	3-oxo-C12-AHL	C7 HQ
t_0_	6.90	0.52	14.1	4.70	0.51	3.21
t_2h_	6.40	nd	10.1	3.70	0.55	4.17
t_24h_	6.10	nd	5.70	3.80	0.49	1.99
	MOF#3			MOF#4		
	C4-AHL	3-oxo-C12-AHL	C7 HQ	C4-AHL	3-oxo-C12-AHL	C7 HQ
t_0_	5.40	0.56	2.85	5.80	0.56	2.85
t_2h_	5.00	0.54	2.75	5.20	0.55	2.75
t_24h_	4.60	0.51	2.50	5.70	0.52	2.50
	MOF#5			MOF#6		
	C4-AHL	3-oxo-C12-AHL	C7 HQ	C4-AHL	3-oxo-C12-AHL	C7 HQ
t_0_	6.70	0.56	17.2	5.10	0.55	4.52
t_2h_	6.00	0.53	12.1	4.00	0.51	3.96
t_24h_	5.90	0.52	5.09	5.50	nd	4.16

## Discussion

### Method validation discussion

The three acquisition techniques showed different LLOQ values and linearity ranges, and MRM was the one which allowed the wider calibration range, with an LLOQ equal to 0.4 μg/L (for PI and NL, LLOQ was 1 μg/L and 5 μg/L, respectively). It is known that the MRM approach has a better sensitivity with respect to NL and PI [46], moreover balanced by the better diagnostic possibilities of these different approaches.

Independently from the MS/MS, the class of the targeted molecule and the calibration range, the heteroscedasticity tests showed concordant results. Both the *F*-test and the Levene test on the variance of the calibration replicates demonstrated the need for a 1/x^2^ weight. The only exceptions are the NL and PI experiments performed for C4-AHL, for which the Levene test suggested a homoscedastic trend. This was discordant with the *t*-test output and also with the visual inspection of the replicates, evidencing a progressively greater variance for replicates of the higher calibration levels. Hence, a more conservative approach was preferred and a weight applied. Furthermore, the verification of the linearity trend by means of a partial *F*-test confirmed the linearity within the selected calibration range, with all the *p* values above the cut-off limit of 0.05. The goodness of the selected calibration model was finally verified by high *p* values for the study of the normality of the standardized residuals and the back-calculation at all the calibration levels (ESM Tables S5 and S6).

### Sample results discussion

**Fig. 4:**
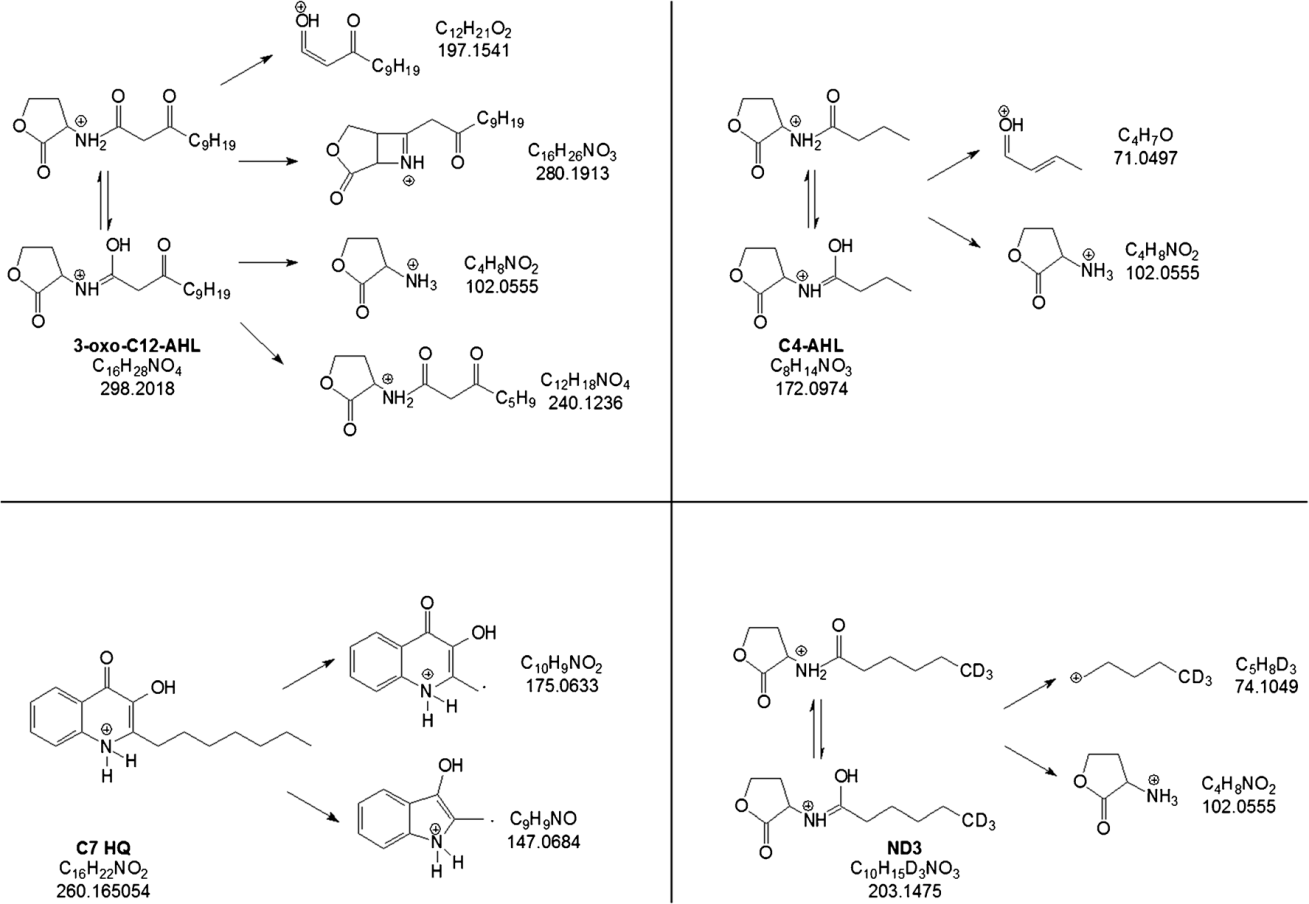
MS^2^ fragmentation pathways of N-(3-oxododecanoyl)-L-homoserine lactone (3-oxo-C12-AHL), N-butanoyl-L-homoserine lactone (C4-AHL), 2-heptyl-3-hydroxy-4(1H)-quinolone (C7 HQ) and N-hexanoyl-L-homoserine lactone-D3 (ND3). Product ion *m/z* 102 and product radical ion *m/z* 175 are the characteristic fragmentation products of molecules belonging to AHL and HQ families, respectively

Before starting with the identification and characterization of unknown AHL and HQ molecules (presented in the section “Sample results” and in ESM Tables S1 and S2), an investigation using HRMS of fragmentation pathways of analytical standards was carried out. As representatives of the AHL class, we selected 3-oxo-C12-AHL and C4-AHL, and C7 HQ from the HQ family. ND3 as previously described was used as IS. CID MS^2^ fragmentation schemes of C4-AHL, 3-oxo-C12-AHL, C7 HQ and ND3 are presented in Fig. 4. As mentioned before, AHL molecules shared the product ion *m/z* 102, and C7 HQ showed the peculiar product radical ion *m/z* 175 common for the HQ family.

Since the analysis of bacterial cultures and plasma samples of patients were completed only 2 days after sampling and handling, and samples were stored refrigerated at −4 °C, no other pathogens should develop [33]. In this situation, the unknown AHLs and HQs detected with the developed MS methods resulted from *P. aeruginosa* bacteria or patients with sepsis.

The chromatographic runs proposed here for AHL molecules were much longer if compared with other analytical methods. Ortoni and co-workers [27] used a 2.9-min-long separation run to quantify 26 AHLs in bacterial cultures. Readel et al. [36], with an acetonitrile isocratic run of 10 min, successfully separated six racemic L/D homoserine lactones. Struss et al. [29] and Hoang et al. [35] developed a 17-min reversed-phase HPLC method to quantify AHL in sputum of cystic fibrosis patients and bacterial cultures, respectively. In particular, the group led by Hoang [35] presented a green analytical method with the use of supercritical-fluid chromatography (SFC), and performed an in-depth optimization of the method. Unfortunately, SFC-TOF (Time of Flight) is quite an expensive technology and requires a very skilled operator. Although these methods showed a very low limit of quantitation (pg/mL), with good selectivity and robustness, they quantified well-known molecules belonging to the AHL family (using analytical standards) [27, 35, 36] or a limited number of analytes [29].

When the number of molecules increases and, more significantly, when unknown compounds should be identified and characterized, the duration of the separation run is increased in order to avoid analyte co-elution or ion suppression. The developed chromatographic run lasted 35 min, which is comparable to that proposed by Kumari et al. [24] for the detection of ten AHLs. An untargeted high-resolution mass spectrometry method for the identification of novel AHLs in eight different bacterial cultures grown under different conditions was proposed by Patel et al. [32]. They used a C18 column and a Q Exactive high-resolution mass spectrometer, and within 13 min they detected 23 AHLs, some of them never described in the literature. Their method showed little overlapping and tailing of chromatographic peaks; nevertheless, they achieved a very low limit of detection for all analytes and performed the identification of unexpected AHL molecules using a labelled approach. As with our method, the MS/MS method for the identification of AHLs was based on a peculiar product ion with *m/z* 102.

The HQ separation run was shorter than that for AHLs, and could be compared to those developed by Maurer et al. [28] and Brewer et al. [34]. It lasted 12 min (Maurer 9′ and Brewer 8′), and the separation obtained with the use of 2-picolinic acid was satisfactory (ESM Fig. S2). Although Maurer and co-workers [28] used a mixture of acetic anhydride-pyridine to derivatize C7-HQ, and the Brewer team [34] employed EDTA as mobile aqueous phase, we preferred to follow the Turnpenny recommendation [33] in order to obtain a good shape of chromatographic peaks and consequently a possibly more accurate quantification of the HQ compounds. Therefore, we ran the chromatographic separation with the use of 2-picolinic acid. Indeed, the EDTA molecule is not suggested for MS analysis, since it is a non-volatile salt and gives an ion suppression effect, and derivatization is not a quantitative operation. On the contrary, 2-picolinic acid is highly volatile and a bidentate chelating agent, and as Turpnenny noted, the use of formic acid together with 2-picolinic acid improved the ESI positive ionization of the studied molecules [33].

As previously presented, we were able to detect and identify 13 HQs in bacterial cultures and plasma samples. Among all the possible QS molecules, the most highly expressed (high concentration) was the C7 HQ. In minimal medium poor in nutrients (W9), the C7 HQ species was the only one detected in both the wild-type and the RhlI derivative bacterial cultures. This finding supports the hypothesis that the knockout mutant, unable to produce C4-AHL, intensified the production of this signal molecule to promote bacterial cell growth. As for AHL compounds, cells grown in LB broth showed the most abundant content of HQ molecules with respect to M9 medium. The detection of HQ molecules in biological samples is very important because their concentration is related to the expression of many virulence factors and to the regulation of iron usage inside the cells [47].

In addition to the importance of a good chromatographic separation, the developed NL and PI MS approaches also played a fundamental role in the identification of unknown compounds. The unknown species C6-AHL was detected in bacterial cultures only, with an empirical formula of C_10_H_17_NO_3_, and accurate *m/z* of 200.1287 (confirmed with the LTQ-Orbitrap system) is in agreement with Alayande et al. [48]. The authors grew *Pseudomonas aeruginosa* PAO1 in LB medium and evaluated the levels of AHL by LC-MS/MS analysis. This work is among the very few on *P. aeruginosa* that showed the detection of C6-AHL signal molecule production, and the only report where C6-AHL was detected in an amount greater than that of 3-oxo-C12-AHL. They found for the first time that among the various signal molecules, C4-AHL and C6-AHL played the most important signalling role in the QS system of *P. aeruginosa* PAO1. Moreover, the search for C6-AHL showed the presence of three chromatographic peaks presumably corresponding to three isomers. Even the known C4-AHL molecule (C_8_H_13_NO_3_, *m/z* 172.0974) was detectable as two different isomers when monitored using the NL approach. However, the ability to discriminate between the isomers would require a more detailed investigation using for example an NMR technique to obtain structural information. The analysis of HQ molecules did not show any isomeric species.

Also, 3-oxo-C10-AHL was recognized in bacterial cultures, and its amount was rather high, especially in RhlI-W9 samples. This indicates that in the absence of C4-AHL, bacteria can use this other molecule to communicate. Similar results were obtained by Patel et al. [32] in *Erwinia carotovora* culture grown in nutrient-rich medium.

Last but not least, we quantified C12-AHL in bacterial samples, to our knowledge for the first time. In fact, other research groups discovered novel AHL molecules in bacterial cultures [27, 32, 35]. For example, Patel and co-workers [32] measured 24 AHLs in eight bacterial cultures using a high-resolution MS approach; they recognized C12-AHL in others cultures, but not in *Pseudomonas aeruginosa*. The same was found for the study by Hoang et al. [35] that quantified C12-AHL in the Gram-negative endophytic bacterium *Paraburkholderia* spp. As previously underlined, C7 HQ, 3-oxo-C10-AHL and finally, C12-AHL could be the alternative controlled factors expressed by mutant *P. aeruginosa* bacteria responsible for their high tolerance to photodynamic therapy and photo-oxidative stress induced by the therapy. These results lay the foundations for more in-depth studies on the *lasI* and the *rhlI* QS signal systems.

An accurate quantitation of the molecules was achieved for bacterial cultures WT-W9, WT-LB, RhlI-W9 and RhlI-LB, and for human plasma samples using the NL and PI validated MS method. We decided to validate the analytical method in plasma QS-free matrix (belonging to healthy volunteers) because the ultimate goal of the study was to quantify QS molecules in plasma of patients affected by MOF. Many other research groups have validated their analytical approaches in bacterial cultures [28, 34, 35]. A comprehensive method for QS compound quantitation in human samples is lacking [23–25, 29]. Furthermore, we studied the *Pseudomonas aeruginosa* QS molecule production because this is one of the most common bacteria found to infect patients with sepsis [49, 50]. Data shown in Table 6 indicate that C4-AHL was detectable in all the samples within a concentration range of 4–7 μg/L, 3-oxo-C12-AHL in a range of 0–0.6 μg/L, and finally, C7-HQ in a range between 2 and 18 μg/L. A general tendency toward a decrease in QS molecule concentration was evidenced, showing that hemoperfusion of plasma of patients with MOF was effective in the removal of QS compounds. The monitoring of QS analytical standard molecules in hemoperfused plasma samples from patients with MOF confirmed the ability of the MRM approach to quantify virulence factors during sepsis with good sensitivity.

## Conclusions

In conclusion, selective, sensitive and robust HPLC-TQ MS methods were developed and applied to wild-type and mutant *P. aeruginosa* bacterial cultures and to biological plasma samples. Thanks to untargeted NL and PI and targeted MRM methods, different AHL and HQ molecules were identified, characterized and quantified.

The HPLC-TQ MS/MS analytical procedure was validated in MRM, NL and PI scan mode. Experimental data indicated that the method was suitable for the detection of low concentrations of AHLs and HQs in bacterial cultures of *Pseudomonas aeruginosa* and biofluids in early stages of sepsis-related multi-organ failure. Using the NL and PI scan methods, it was possible to discover a new kind of species whose presence within the sample was not predictable at the beginning of the analysis. The presence of high concentrations of C7 HQ, 3-oxo-C10-AHL and C12-AHL in mutant strains of *P. aeruginosa* knocked for the production of C4-AHL could be the starting point for a better understanding of how bacteria exploit the controlled factors to survive and proliferate. The MRM approach was suitable for the detection of low AHL and HQ levels within less abundant samples. The comparison between bacterial cultures and plasma samples highlighted even more why high-sensitivity methods are essential for plasma, given the concentration of a few micrograms per litre of the molecules of interest within the sample.

The three validated approaches were demonstrated to be reproducible, repeatable, robust and sensitive enough to quantify QS in real biological samples and, we think, to have evidenced the possibility of applying them for solving practical analytical problems with known limitations of sensitivity. The LTQ-Orbitrap-HRMS platform has been confirmed as an indispensable tool for investigating the fragmentation pathways and confirming the detection of unexpected QS molecules.

In an on-going study in patients with MOF, we are demonstrating the ability of hemoperfusion to reduce the QS amount, underlining the physiopathological implications of these findings. Here we have presented the usefulness of the studied LC-MS approaches and applied them to real samples.

## Supplementary Information

ESM 1 (PDF 512 kb)

## Data Availability

The data reported in this manuscript that support the findings of this study are available on request from the corresponding author on reasonable request. The data are not publicly available as they contain patients’ personal information that could compromise their privacy.

## Abbreviations

3-oxo-C12-AHL: N-(3-Oxododecanoyl)-L-homoserine lactone
AHLs: N-Acyl homoserine lactones
AI: Autoinducer
C4-AHL: N-Butanoyl-L-homoserine lactone
C7 HQ (or PQS): 2-Heptyl-3-hydroxy-4(1H)-quinolone
HPLC: High-performance liquid chromatography
HPLC-TQ MS: High-performance liquid chromatography–triple quadrupole mass spectrometry
HRMS: High-resolution mass spectrometry
HQs: Hydroxyquinolone signalling molecules
IS: Internal standard
LB: Luria-Bertani
LLOQ: Lower limit of quantification
LTQ: Linear trap quadrupole
M9: Mineral medium
MOF: Multi-organ failure
MRM: Multiple reaction monitoring
MS: Mass spectrometry
MW: Molecular weight
NL: Neutral loss
PI: Product ion
RT: Room temperature
QS: Quorum sensing
TQ MS: Triple quadrupole mass spectrometer
UHPLC: Ultra-high-performance liquid chromatography
ULOQ: Upper limit of quantification

## References

[1] Turovskiy Y, Kashtanov D, Paskhover B, Chikindas ML (2007). Quorum sensing: fact, fiction, and everything in between. Adv Appl Microbiol.

[2] Abisado RG, Benomar S, Klaus JR, Dandekar AA, Chandler JR (2018). Bacterial quorum sensing and microbial community interactions. mBio.

[3] Mukherjee S, Bassler BL (2019). Bacterial quorum sensing in complex and dynamically changing environments. Nat Rev Microbiol.

[4] Rajeshkannan E, Saini S. Physiological advantage of phenotypic heterogeneity in a quorum-sensing population. J Indian Inst Sci. 2020; 100:485–96. 10.1007/s41745-020-00175-4.

[5] Leichnitz D, Raguž L, Beemelmanns C (2017). Total synthesis and functional analysis of microbial signaling molecules. Chem Soc Rev.

[6] Hense BA, Schuster M (2015). Core principles of bacterial autoinducer systems. Microbiol Mol Biol Rev.

[7] de Kievit TR, Iglewski BH (2000). Bacterial quorum sensing in pathogenic relationships. Infect Immun.

[8] Diggle SP, Griffin AS, Campbell GS, West SA (2007). Cooperation and conflict in quorum-sensing bacterial populations. Nature..

[9] Pai A, Tanouchi Y, You L (2012). Optimality and robustness in quorum sensing (QS)-mediated regulation of a costly public good enzyme. Proc Natl Acad Sci U S A.

[10] Paul D, Gopal J, Kumar M, Manikandan M (2018). Nature to the natural rescue: silencing microbial chats. Chem Biol Interact.

[11] Galloway WR, Hodgkinson JT, Bowden SD, Welch M, Spring DR (2011). Quorum sensing in gram-negative bacteria: small-molecule modulation of AHL and AI-2 quorum sensing pathways. Chem Rev.

[12] Cataldi TR, Bianco G, Fonseca J, Schmitt-Kopplin P (2013). Perceiving the chemical language of gram-negative bacteria: listening by high-resolution mass spectrometry. Anal Bioanal Chem.

[13] Chbib C (2020). Impact of the structure-activity relationship of AHL analogues on quorum sensing in gram-negative bacteria. Bioorg Med Chem.

[14] Dickschat JS (2010). Quorum sensing and bacterial biofilms. Nat Prod Rep.

[15] Prescott RD, Decho AW (2020). Flexibility and adaptability of quorum sensing in nature. Trends Microbiol.

[16] Pearson JP, Pesci EC, Iglewski BH (1997). Roles of *Pseudomonas aeruginosa las* and *rhl* quorum-sensing systems in control of elastase and rhamnolipid biosynthesis genes. J Bacteriol.

[17] Soukarieh F, Williams P, Stocks MJ, Cámara M (2018). *Pseudomonas aeruginosa* quorum sensing systems as drug discovery targets: current position and future perspectives. J Med Chem.

[18] Lin J, Cheng J, Wang Y, Shen X (2018). The Pseudomonas quinolone signal (PQS): not just for quorum sensing anymore. Front Cell Infect Microbiol.

[19] García-Reyes S, Soberón-Chávez G, Cocotl-Yanez M (2020). The third quorum-sensing system of *Pseudomonas aeruginosa*: *Pseudomonas* quinolone signal and the enigmatic PqsE protein. J Med Microbiol.

[20] Dong YH, Wang LH, Xu JL, Zhang HB, Zhang XF, Zhang LH (2001). Quenching quorum-sensing-dependent bacterial infection by an N-acyl homoserine lactonase. Nature.

[21] Diggle SP, Winzer K, Chhabra SR, Worrall KE, Cámara M, Williams P (2003). The *Pseudomonas aeruginosa* quinolone signal molecule overcomes the cell density-dependency of the quorum sensing hierarchy, regulates rhl-dependent genes at the onset of stationary phase and can be produced in the absence of LasR. Mol Microbiol.

[22] Steindler L, Venturi V (2007). Detection of quorum-sensing N-acyl homoserine lactone signal molecules by bacterial biosensors. FEMS Microbiol Lett.

[23] Middleton B, Rodgers HC, Cámara M, Knox AJ, Williams P, Hardman A (2002). Direct detection of N-acylhomoserine lactones in cystic fibrosis sputum. FEMS Microbiol Lett.

[24] Kumari A, Pasini P, Daunert S (2008). Detection of bacterial quorum sensing N-acyl homoserine lactones in clinical samples. Anal Bioanal Chem.

[25] Campagna SR, Gooding JR, May AL (2009). Direct quantitation of the quorum sensing signal, autoinducer-2, in clinically relevant samples by liquid chromatography-tandem mass spectrometry. Anal Chem.

[26] Massai F, Imperi F, Quattrucci S, Zennaro E, Visca P, Leoni L (2011). A multitask biosensor for micro-volumetric detection of N-3-oxo-dodecanoyl-homoserine lactone quorum sensing signal. Biosens Bioelectron.

[27] Ortori CA, Dubern JF, Chhabra SR, Cámara M, Hardie K, Williams P, Barrett DA (2011). Simultaneous quantitative profiling of N-acyl-L-homoserine lactone and 2-alkyl-4(1H)-quinolone families of quorum-sensing signaling molecules using LC-MS/MS. Anal Bioanal Chem.

[28] Maurer CK, Steinbach A, Hartmann RW (2013). Development and validation of a UHPLC-MS/MS procedure for quantification of the Pseudomonas quinolone signal in bacterial culture after acetylation for characterization of new quorum sensing inhibitors. J Pharm Biomed Anal.

[29] Struss AK, Nunes A, Waalen J, Lowery CA, Pullanikat P, Denery JR, Conrad DJ, Kaufmann GF, Janda KD (2013). Toward implementation of quorum sensing autoinducers as biomarkers for infectious disease states. Anal Chem.

[30] Nasuno E, Okano C, Iimura K, Morohoshi T, Ikeda T, Kato N (2014). Quick detection of cell to cell communication in gram negative bacteria by colour change of polymer matrix entrapping reporter bacteria. Materials Res Innov..

[31] Charlesworth J, Kimyon O, Manefield M, Burns BP (2015). Detection and characterization of N-acyl-l-homoserine lactones using GFP-based biosensors in conjunction with thin-layer chromatography. J Microbiol Methods.

[32] Patel NM, Moore JD, Blackwell HE, Amador-Noguez D (2016). Identification of unanticipated and novel N-acyl L-Homoserine lactones (AHLs) using a sensitive non-targeted LC-MS/MS method. PLoS One.

[33] Turnpenny P, Padfield A, Barton P, Teague J, Rahme LG, Pucci MJ, Zahler R, Rubio A (2017). Bioanalysis of *Pseudomonas aeruginosa* alkyl quinolone signaling molecules in infected mouse tissue using LC-MS/MS; and its application to a pharmacodynamic evaluation of MvfR inhibition. J Pharm Biomed Anal.

[34] Brewer LK, Jones JW, Blackwood CB, Barbier M, Oglesby-Sherrouse A, Kane MA (2020). Development and bioanalytical method validation of an LC-MS/MS assay for simultaneous quantitation of 2-alkyl-4(1H)-quinolones for application in bacterial cell culture and lung tissue. Anal Bioanal Chem.

[35] Hoang TPT, Barthélemy M, Lami R, Stien D, Eparvier V, Touboul D (2020). Annotation and quantification of N-acyl homoserine lactones implied in bacterial quorum sensing by supercritical-fluid chromatography coupled with high-resolution mass spectrometry. Anal Bioanal Chem.

[36] Readel E, Portillo A, Talebi M, Armstrong DW (2020). Enantiomeric separation of quorum sensing autoinducer homoserine lactones using GC-MS and LC-MS. Anal Bioanal Chem.

[37] Orlandi VT, Bolognese F, Chiodaroli L, Tolker-Nielsen T, Barbieri P (2015). Pigments influence the tolerance of *Pseudomonas aeruginosa* PAO1 to photodynamically induced oxidative stress. Microbiology..

[38] Orlandi VT, Bolognese F, Martegani E, Cantaluppi V, Medana C, Barbieri P (2017). Response to photo-oxidative stress of *Pseudomonas aeruginosa* PAO1 mutants impaired in different functions. Microbiology.

[39] Wang J, Quan C, Wang X, Zhao P, Fan S (2011). Extraction, purification and identification of bacterial signal molecules based on N-acyl homoserine lactones. Microb Biotechnol.

[40] European Medicines Agency. ICH Topic Q 2 (R1) Validation of analytical procedures: text and methodology. CPMP/ICH/381/95. 2005. Publisher: ICH Secretariat, c/o IFPMA, 30 rue de St - Jean, P.O. Box 758, 1211 Geneva 13, Switzerland

[41] Eurachem (2017). Bioanalytical Method Validation Guidance. J Chromatogr B Anal Technol Biomed Life Sci [Internet].

[42] Alladio E, Amante E, Bozzolino C, Seganti F, Salomone A, Vincenti M, Desharnais B (2020). Effective validation of chromatographic analytical methods: the illustrative case of androgenic steroids. Talanta..

[43] Desharnais B, Camirand-Lemyre F, Mireault P, Skinner CD (2017). Procedure for the selection and validation of a calibration model i-description and application. J Anal Toxicol.

[44] Desharnais B, Camirand-Lemyre F, Mireault P, Skinner CD (2017). Procedure for the selection and validation of a calibration model ii-theoretical basis. J Anal Toxicol.

[45] Alladio E, Amante E, Bozzolino C, Seganti F, Salomone A, Vincenti M, Desharnais B (2020). Experimental and statistical protocol for the effective validation of chromatographic analytical methods. MethodsX..

[46] El-Aneed A, Cohen A, Banoub T (2009). Mass spectrometry, review of the basics: electrospray, maldi, and commonly used mass analyzers. Appl Spectrosc Rev.

[47] Bredenbrusch F, Geffers R, Nimtz M, Beur J, Haüsler S (2006). The *Pseudomonas aeruginosa* quinolone signal (PQS) has an iron-chelating activity. Environ Microbiol.

[48] Alayande AB, Aung MM, Kim IS (2018). Correlation between quorum sensing signal molecules and *Pseudomonas aeruginosa*’s biofilm development and virulency. Curr Microbiol.

[49] Vincent JL, Bihari DJ, Suter PM, Bruining HA, White J, Nicolas-Chanoin MH, Wolff M, Spencer RC, Hemmer M (1995). The prevalence of nosocomial infection in intensive care units in Europe. Results of the European prevalence of infection in intensive care (EPIC) study. EPIC international advisory committee. JAMA..

[50] Vincent JL, Sakr Y, Singer M, Martin-Loeches I, Machado FR, Marshall JC, Finfer S, Pelosi P, Brazzi L, Aditianingsih D, Timsit JF, Du B, Wittebole X, Máca J, Kannan S, Gorordo-Delsol LA, De Waele JJ, Mehta Y, Bonten MJM, Khanna AK, Kollef M, Human M, Angus DC, Investigators EPICIII (2020). Prevalence and Outcomes of Infection Among Patients in Intensive Care Units in 2017. JAMA.

