# Supplementary material for: Targeted and untargeted quantification of quorum sensing signalling molecules in bacterial cultures and biological samples via HPLC-TQ MS techniques
Source: Anal Bioanal Chem. 2020 Nov 18;413(3):853–64. doi: 10.1007/s00216-020-03040-6 (PMC7809007; doi:10.1007/s00216-020-03040-6)
Supplement: Supplementary file 1 — (PDF 512 kb) [file 216_2020_3040_MOESM1_ESM.pdf]

Analytical and Bioanalytical Chemistry

Electronic Supplementary Material

Targeted and untargeted quantification of quorum sensing signalling molecules in bacterial cultures and biological samples via HPLC-TQ MS techniques

Federica Dal Bello, Michael Zorzi, Riccardo Aigotti, Davide Medica, Vito Fanelli, Vincenzo Cantaluppi, Eleonora Amante, Viviana Teresa Orlandi, Claudio Medana

1. Figures

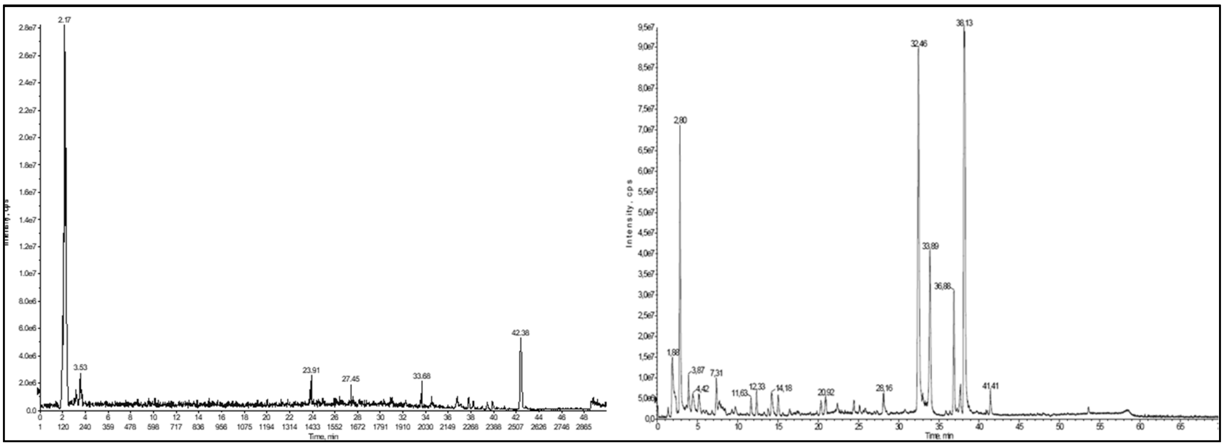

Fig. S1 Chromatograms acquired with NL MS approach for AHLs signalling molecules analysis. On the left it was presented the separation of few AHL compounds in a sample of patient plasma. As discussed in the main manuscript, since the sample was poor in AHL detection, the elution gradient was of 48 minutes and no overlapping peaks were observed. On the contrary, the right panel shows the NL AHLs separation in a sample of *Pseudomonas aeruginosa* wild type grown in Luria Bertani (LB) broth. Here, many AHL molecules were detected and the gradient separation was slower compared with plasma sample in order to obtain a satisfactory separation of peaks

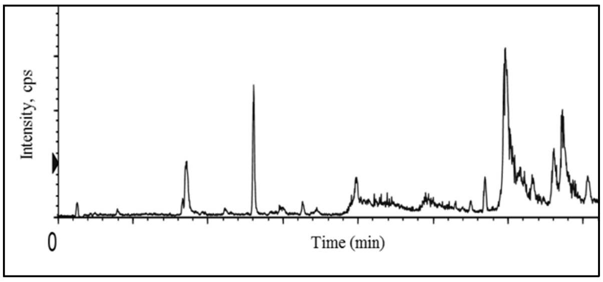

Fig. S2 Chromatogram acquired with PI MS approach for HQs signalling molecules analysis using 2-picolinic acid as aqueous mobile phase

2. Validation tables and results

*The acronym AUC means “area under the curve”.

2.1. 3-oxo-C12-AHL (N-(3-oxododecanoyl)-L-homoserine lactone)

2.1.1. Calibration curves AUC and equation

-MRM

Conc. (µg/L)	AUC cal. curve 1	AUC cal. curve 2	AUC cal. curve 3	AUC cal. curve 4
0.4	4.91E+04	4.81E+04	6.58E+04	5.75E+04
1	1.12E+05	1.19E+05	1.60E+05	1.40E+05
5	6.21E+05	7.30E+05	9.11E+05	5.80E+05
10	1.27E+06	1.48E+06	1.88E+06	1.12E+06
50	6.46E+06	7.94E+06	1.00E+07	7.06E+06
100	1.07E+07	1.30E+07	1.62E+07	1.25E+07
200	2.30E+07	2.84E+07	3.52E+07	2.67E+07
300	3.56E+07	4.25E+07	5.04E+07	40117103
400	4.56E+07	5.57E+07	6.81E+07	5.41E+07

Linear regressive analysis using a weighting factor of $1/x^2$: y= 141900x-2609

-NL

Conc. (µg/L)	AUC cal. curve 1	AUC cal. curve 2	AUC cal. curve 3	AUC cal. curve 4
5	2.67E+06	2.72E+06	2.69E+06	2.72E+06
10	4.12E+06	4.52E+06	4.88E+06	4.99E+06
50	2.31E+07	2.65E+07	2.80E+07	2.95E+07
100	4.03E+07	5.12E+07	4.83E+07	4.79E+07
200	9.90E+07	9.17E+07	9.99E+07	1.08E+08
300	1.41E+08	1.40E+08	1.41E+08	1.45E+08
400	1.90E+08	1.98E+08	2.14E+08	2.14E+08

Linear regressive analysis using a weighting factor of $1/x^2$: y= 490900x + 152400

-PI

Conc. (µg/L)	AUC cal. curve 1	AUC cal. curve 2	AUC cal. curve 3	AUC cal. curve 4
1	8.40E+05	8.57E+05	9.19E+05	6.66E+05
5	3.55E+06	7.69E+05	4.71E+06	4.13E+06
10	7.33E+06	5.67E+06	7.45E+06	6.54E+06
50	3.47E+07	3.59E+07	40318826	3.59E+07
100	7.27E+07	6.98E+07	78447022	7.07E+07
200	1.41E+08	1.37E+08	1.54E+08	1.50E+08
300	1.83E+08	2.30E+08	2.58E+08	2.25E+08

Linear regressive analysis using a weighting factor of $1/x^2$: y= 709400x + 93520

2.1.2. Selectivity (SEL%)

- MRM

**Sample**	**AUC**	**Average AUC**	**SEL%**	**8.12**
QS-free_matrix_001	3.45E+03			
QS-free_matrix_002	4.67E+03	4.41E+03		
QS-free_matrix_003	5.12E+03			
STD_400 ppt_001	4.91E+04			
STD_400 ppt_002	4.81E+04	5.43E+04		
STD_400 ppt_003	6.58E+04			

-NL

**Sample**	**AUC**	**Average AUC**	**SEL%**	**0.50**
QS-free_matrix_001	1.14E+04			
QS-free_matrix_002	1.54E+04	1.34E+04		
QS-free_matrix_003	1.33E+04			
STD_5 ppb_001	2.67E+06			
STD_5 ppb_002	2.72E+06	2.69E+06		
STD_5 ppb_003	2.69E+06			

-PI

**Sample**	**AUC**	**Average AUC**	**SEL%**	**1.10**
QS-free_matrix_001	9.94E+03			
QS-free_matrix_002	8.77E+03	9.58E+03		
QS-free_matrix_003	1.00E+04			
STD_1 ppb_001	8.40E+05			
STD_1 ppb_002	8.57E+05	8.72E+05		
STD_1 ppb_003	9.19E+05			

2.1.3. Recovery (REC%)

- MRM

Sample	AUC	Average AUC
STD_0.4ppb_solv_01	1.26E+05	1.09E+05
STD_0.4ppb_solv_02	9.87E+04	
STD_0.4ppb_solv_03	1.02E+05	
STD_400ppb_solv_01	1.02E+08	9.87E+07
STD_400ppb_solv_02	9.74E+07	
STD_400ppb_solv_03	9.63E+07	
STD_0.4ppb_pls_01	5.00E+04	4.95E+04
STD_0.4ppb_pls_02	5.00E+04	

STD_0.4ppb_pls_03	4.86E+04	
STD_400ppb_pls_01	5.34E+07	5.30E+07
STD_400ppb_pls_02	5.54E+07	
STD_400ppb_pls_03	5.03E+07	
**REC%**	**LLOQ**	**45.5**
	**ULOQ**	**53.7**

2.1.4. Carry-over (CO%)

	Sample	AUC		
MRM	STD_400 ppb	4.56E+07	**CO%**	**7.10**
	QS-free_matrix	4.67E+03		
	STD_400 ppt	6.58E+04		
NL	STD_400 ppb	1.90E+08	**CO%**	**0.57**
	QS-free_matrix	1.54E+04		
	STD_5 ppb	2.69E+06		
PI	STD_300 ppb	1.83E+08	**CO%**	**1.29**
	QS-free_matrix	9.94E+03		
	STD_1 ppb	7.69E+05		

2.1.5. Intra-day accuracy (BIAS%) and precision (CV%)

-MRM

Nominal conc. (µg/L)	Real conc. (back-calculated, µg/L)				CV%	BIAS%
	Cal. curve 1	Cal. curve 2	Cal. curve 3	Cal. curve 4		
0.4	0.36	0.36	0.48	0.42	12.4	1.65
1	0.81	0.85	1.15	1.00	14.0	4.92
5	4.39	5.16	6.44	4.11	18.0	0.50
10	9.00	10.5	13.3	7.88	20.0	1.51
50	45.5	56.0	70.6	49.8	17.1	9.88
100	75.5	91.8	114	88.2	15.1	8.16
200	162	200	248	189	15.6	0.15
300	251	300	355	283	12.8	0.96
400	322	392	480	381	14.3	1.61

-NL

Nominal conc. (µg/L)	Real conc. (back-calculated, µg/L)				CV%	BIAS%
	Cal. curve 1	Cal. curve 2	Cal. curve 3	Cal. curve 4		
5	5.13	5.23	5.17	5.24	0.87	3.66
10	8.07	8.90	9.63	9.85	7.64	9.72
50	46.8	53.6	56.6	59.8	8.87	7.77
100	81.8	104	98.0	97.2	8.60	4.99
200	201	187	203	221	5.96	1.45
300	287	284	287	296	1.53	4.00
400	387	404	435	435	4.98	3.63

-PI

Nominal conc. (µg/L)	Real conc. (back-calculated, µg/L)				CV%	BIAS%
	Cal. curve 1	Cal. curve 2	Cal. curve 3	Cal. curve 4		
1	1.05	1.08	1.16	0.81	12.9	2.40
5	4.88	0.95	6.51	5.69	47.3	10.9
10	10.2	7.86	10.4	9.09	10.8	6.63
50	48.7	50.5	56.7	50.4	5.91	3.05
100	102	98.3	111	99.5	4.62	2.56
200	199	193	217	211	4.62	2.51
300	258	324	364	317	12.0	4.97

2.1.6. LOD and LOQ

-MRM

Sample	AUC		
QS-free_matrix_001	3.45E+03		
QS-free_matrix_002	4.67E+03		
QS-free_matrix_003	5.12E+03		
QS-free_matrix_004	5.02E+03		
QS-free_matrix_005	4.11E+03	Slope	1.42E+05
QS-free_matrix_006	4.53E+03	Intercept	-2.61E+03
Average AUC	4.48E+03		
Std. dev QS-free matrix	5.69E+02		**Conc. (µg/L)**
10std. Dev. + Average AUC	1.02E+04	**LOQ**	**0.090**

-NL

Sample	AUC		
QS-free_matrix_001	4.58E+03		
QS-free_matrix_002	4.69E+03		
QS-free_matrix_003	4.82E+03		
QS-free_matrix_004	3.52E+03		
QS-free_matrix_005	4.11E+03	Slope	4.91E+05
QS-free_matrix_006	4.53E+03	Intercept	1.52E+05
Average AUC	4.37E+03		
Std. dev QS-free matrix	4.42E+02		**Conc. (µg/L)**
10std. Dev. + Average AUC	8.80E+03	**LOQ**	**0.293**

-PI

Sample	AUC		
QS-free_matrix_001	3.45E+03		
QS-free_matrix_002	4.67E+03		
QS-free_matrix_003	5.12E+03		
QS-free_matrix_004	5.02E+03		
QS-free_matrix_005	4.11E+03	Slope	7.09E+05
QS-free_matrix_006	4.53E+03	Intercept	9.35E+04
Average AUC	4.48E+03		
Std. dev QS-free matrix	5.69E+02		**Conc. (µg/L)**
10std. Dev. + Average AUC	1.02E+04	**LOQ**	**0.117**

2.1.7. Freeze-thaw stability (STAB%)

-MRM

	AUC T_0	AUC $T_{freeze\text{-}thaw}$	Conc. (µg/L) T_0	Conc. (µg/L) $T_{freeze\text{-}thaw}$	STAB%	AVAREGE STAB%
LLOQ (0.4 µg/L)	1.23E+05	1.23E+05	0.88	0.88	100	106
	5.54E+04	4.81E+04	0.41	0.36	115	
	5.43E+04	5.57E+04	0.40	0.41	102	
ULOQ (400 µg/L)	5.54E+07	5.63E+07	390	397	102	101
	5.55E+07	5.58E+07	392	393	100	
	5.60E+07	5.68E+07	394	400	101	

-NL

	AUC T_0	AUC $T_{freeze\text{-}thaw}$	Conc. (µg/L) T_0	Conc. (µg/L) $T_{freeze\text{-}thaw}$	STAB%	AVAREGE STAB%
LLOQ (5 µg/L)	2.78E+06	2.41E+06	5.35	4.60	86.0	89.5
	2.61E+06	2.59E+06	5.01	4.96	98.9	
	2.61E+06	2.21E+06	5.01	4.18	83.5	
ULOQ (400 µg/L)	1.98E+08	1.80E+08	403	367	91.1	88.6
	2.05E+08	1.82E+08	417	371	88.9	
	2.06E+08	1.77E+08	420	360	85.8	

-PI

	AUC T_0	AUC $T_{freeze\text{-}thaw}$	Conc. (µg/L) T_0	Conc. (µg/L) $T_{freeze\text{-}thaw}$	STAB%	AVAREGE STAB%
LLOQ (1 µg/L)	9.88E+05	7.20E+05	1.26	0.88	70.0	86.0
	6.59E+05	6.61E+05	0.80	0.80	100	
	8.14E+05	7.24E+05	1.02	0.89	87.6	
ULOQ (300 µg/L)	2.23E+08	2.13E+08	315	300	95.2	90.2
	1.99E+08	1.86E+08	281	261	93.2	
	1.87E+08	1.54E+08	264	217	82.2	

2.1.8. Figure of average of four calibration curves

-MRM

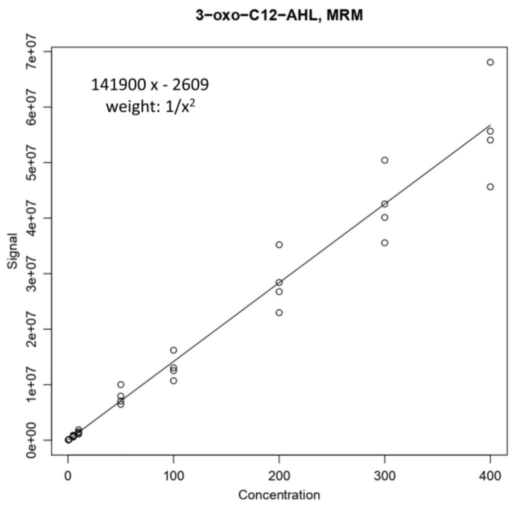

-NL

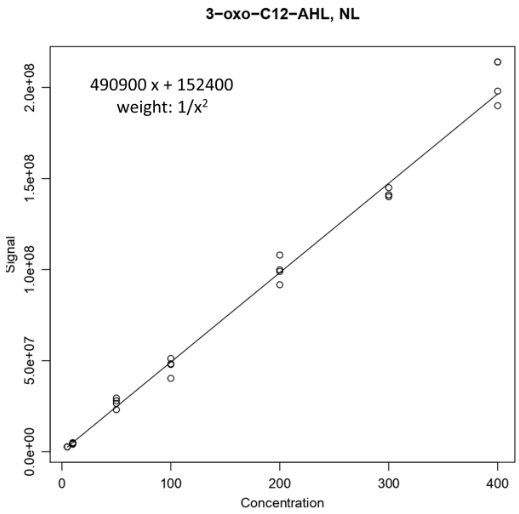
3−oxo−C12−AHL, NL
490900 x + 152400
weight: 1/x²
Signal
Concentration
0.0e+00
5.0e+07
1.0e+08
1.5e+08
2.0e+08
0
100
200
300
400

-PI

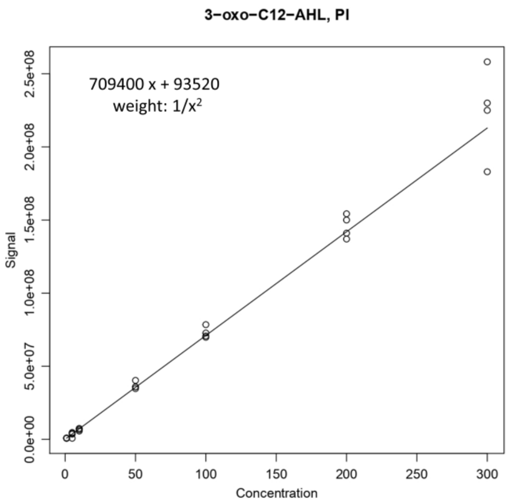
3−oxo−C12−AHL, PI
709400 x + 93520
weight: 1/x²
Signal
Concentration
0.0e+00
5.0e+07
1.0e+08
1.5e+08
2.0e+08
2.5e+08
0
50
100
150
200
250
300

2.2. C4-AHL (N-butanoyl-L-homoserine lactone)

2.2.1. Calibration curves AUC and equation

-MRM

Conc. (µg/L)	AUC cal. curve 1	AUC cal. curve 2	AUC cal. curve 3	AUC cal. curve 4
0.4	5.78E+03	6.18E+03	8.73E+03	7.13E+03
1	1.28E+04	1.40E+04	2.08E+04	1.75E+04
5	9.27E+04	1.00E+05	6.08E+04	8.16E+04
10	1.42E+05	1.48E+05	1.79E+05	1.61E+05
50	7.10E+05	7.58E+05	8.93E+05	9.20E+05
100	1.29E+06	1.44E+06	1.64E+06	1.59E+06
200	2.43E+06	2.89E+06	3.54E+06	2.58E+06
300	3.77E+06	4.18E+06	5.53E+06	5.03E+06
400	4.89E+06	5.68E+06	7.38E+06	6.83E+06

Linear regressive analysis using a weighting factor of $1/x^2$: y= 15520x + 783.5

-NL

Conc. (µg/L)	AUC cal. curve 1	AUC cal. curve 2	AUC cal. curve 3	AUC cal. curve 4
5	1.00E+05	7.95E+04	1.39E+05	1.06E+05
10	1.52E+05	1.52E+05	2.28E+05	1.77E+05
50	7.58E+05	7.22E+05	1.14E+06	8.73E+05
100	1.44E+06	1.40E+06	2.42E+06	1.75E+06
200	2.89E+06	2.91E+06	4.41E+06	3.40E+06
300	4.18E+06	4.11E+06	6.29E+06	4.86E+06
400	5.68E+06	5.55E+06	9.07E+06	6.77E+06

Linear regressive analysis using a weighting factor of $1/x^2$: y= 16740x + 20230

-PI

Conc. (µg/L)	AUC cal. curve 1	AUC cal. curve 2	AUC cal. curve 3	AUC cal. curve 4
1	1.68E+05	1.61E+05	1.16E+05	1.49E+05
5	8.05E+05	1.31E+06	5.64E+05	8.64E+05
10	1.34E+06	2.12E+06	1.24E+06	1.55E+06
50	7.62E+06	8.29E+06	5.21E+06	7.91E+06
100	1.50E+07	1.61E+07	1.11E+07	1.79E+07
200	3.02E+07	2.98E+07	2.22E+07	3.32E+07
300	4.47E+07	4.44E+07	2.99E+07	5.09E+07

Linear regressive analysis using a weighting factor of $1/x^2$: y= 151400x + 2447

2.2.2. Selectivity (SEL%)

- MRM

Sample	AUC	Average AUC	SEL%	5.65
QS-free_matrix_001	4.82E+02			
QS-free_matrix_002	4.85E+02	7.44E+02		
QS-free_matrix_003	1.27E+03			
STD_400 ppt_001	1.88E+04			
STD_400 ppt_002	1.20E+04	1.32E+04		
STD_400 ppt_003	8.73E+03			

-NL

Sample	AUC	Average AUC	SEL%	1.63
QS-free_matrix_001	1.75E+03			
QS-free_matrix_002	1.65E+03	1.76E+03		
QS-free_matrix_003	1.88E+03			
STD_5 ppb_001	7.95E+04			
STD_5 ppb_002	1.39E+05	1.08E+05		
STD_5 ppb_003	1.06E+05			

-PI

Sample	AUC	Average AUC	SEL%	2.54
QS-free_matrix_001	5.94E+03			
QS-free_matrix_002	4.77E+03	5.58E+03		
QS-free_matrix_003	6.03E+03			
STD_1 ppb_001	7.64E+04			
STD_1 ppb_002	1.71E+05	2.20E+05		
STD_1 ppb_003	4.13E+05			

2.2.3. Recovery (REC%)

Sample	AUC	Average AUC
STD_0.4ppb_solv_01	1.59E+04	1.42E+04
STD_0.4ppb_solv_02	1.42E+04	
STD_0.4ppb_solv_03	1.26E+04	
STD_400ppb_solv_01	1.59E+07	1.49E+07
STD_400ppb_solv_02	1.24E+07	
STD_400ppb_solv_03	1.63E+07	
STD_0.4ppb_pls_01	6.45E+03	7.26E+03
STD_0.4ppb_pls_02	7.86E+03	
STD_0.4ppb_pls_03	7.46E+03	
STD_400ppb_pls_01	1.03E+07	9.05E+06
STD_400ppb_pls_02	7.24E+06	
STD_400ppb_pls_03	9.65E+06	
**REC%**	**LLOQ**	**51.0**
	**ULOQ**	**60.9**

2.2.4. Carry-over (CO%)

	**Sample**	**AUC**		
	STD_400 ppb	6.93E+06	**CO%**	**8.90**
MRM	QS-free_matrix	4.82E+02		
	STD_400 ppt	5.41E+03		
	STD_400 ppb	6.77E+06	**CO%**	**1.65**
NL	QS-free_matrix	1.65E+03		
	STD_5 ppb	1.00E+05		
	STD_300 ppb	1.26E+05	**CO%**	**0.87**
PI	QS-free_matrix	1.27E+03		
	STD_1 ppb	1.46E+05		

2.2.5. Intra-day accuracy (BIAS%) and precision (CV%)

-MRM

Nominal conc. (μg/L)	Real conc. (back-calculated, μg/L)				CV%	BIAS%
	Cal. curve 1	Cal. curve 2	Cal. curve 3	Cal. curve 4		
0.4	0.32	0.35	0.51	0.41	18.4	0.61
1	0.77	0.85	1.29	1.08	20.2	0.03
5	5.93	6.39	3.87	5.20	17.8	6.49
10	9.12	9.49	11.5	10.3	8.93	0.96
50	45.7	48.8	57.5	59.2	10.8	5.32
100	83.2	92.4	105	102	9.05	4.43
200	157	186	228	166	14.9	8.57
300	243	270	357	324	15.0	0.61
400	315	366	475	440	15.6	0.23

-NL

Nominal conc. (μg/L)	Real conc. (back-calculated, μg/L)				CV%	BIAS%
	Cal. curve 1	Cal. curve 2	Cal. curve 3	Cal. curve 4		
5	4.76	3.54	7.11	5.14	25.0	2.72
10	7.87	7.87	12.4	9.38	19.7	6.64
50	44.1	41.9	66.8	50.9	19.1	1.84
100	84.5	82.1	143	103	23.7	3.19
200	171	173	262	202	18.2	1.03
300	249	245	375	289	18.1	3.67
400	338	331	541	403	20.9	0.80

-PI

Nominal conc. (μg/L)	Real conc. (back-calculated, μg/L)				CV%	BIAS%
	Cal. curve 1	Cal. curve 2	Cal. curve 3	Cal. curve 4		
1	1.10	1.05	0.75	0.97	13.7	3.59
5	5.30	8.63	3.71	5.69	30.5	14.3
10	8.83	14.0	8.19	10.2	21.8	2.97
50	50.3	54.8	34.4	52.3	16.6	4.32
100	99.3	106	73.4	118	16.6	0.67
200	199	197	147	220	14.0	4.97
300	295	293	198	336	18.1	6.98

2.2.6. LOD and LOQ

-MRM

Sample	AUC		
QS-free_matrix_001	4.82E+02		
QS-free_matrix_002	4.85E+02		
QS-free_matrix_003	1.27E+03		
QS-free_matrix_004	4.72E+02		
QS-free_matrix_005	1.27E+03	Slope	1.55E+04
QS-free_matrix_006	1.36E+03	Intercept	7.84E+02
Average AUC	8.88E+02		
Std. dev QS-free matrix	4.10E+02		**Conc. (µg/L)**
10std. Dev. + Average AUC	4.99E+03	**LOQ**	**0.271**

-NL

Sample	AUC		
QS-free_matrix_001	4.82E+02		
QS-free_matrix_002	4.85E+02		
QS-free_matrix_003	1.27E+03		
QS-free_matrix_004	1.36E+03		
QS-free_matrix_005	3.65E+03	Slope	1.67E+04
QS-free_matrix_006	4.02E+02	Intercept	2.02E+04
Average AUC	1.27E+03		
Std. dev QS-free matrix	1.13E+03		**Conc. (µg/L)**
10std. Dev. + Average AUC	1.26E+04	**LOQ**	**0.457**

-PI

Sample	AUC		
QS-free_matrix_001	1.27E+03		
QS-free_matrix_002	1.36E+03		
QS-free_matrix_003	3.65E+03		
QS-free_matrix_004	4.02E+02		
QS-free_matrix_005	2.47E+02	Slope	1.51E+05
QS-free_matrix_006	1.25E+03	Intercept	2.45E+03
Average AUC	1.36E+03		
Std. dev QS-free matrix	1.11E+03		**Conc. (µg/L)**
10std. Dev. + Average AUC	1.25E+04	**LOQ**	**0.066**

2.2.7. Freeze-thaw stability (STAB%)

-MRM

	AUC T_0	AUC $T_{freeze\text{-}thaw}$	Conc. (µg/L) T_0	Conc. (µg/L) $T_{freeze\text{-}thaw}$	STAB%	AVAREGE STAB%
LLOQ (0.4 µg/L)	7.24E+03	6.59E+03	0.42	0.37	90.0	97.3
	6.72E+03	6.95E+03	0.38	0.40	104	
	7.27E+03	7.13E+03	0.42	0.41	97.9	
ULOQ (400 µg/L)	6.09E+06	6.32E+06	392	407	104	102
	6.16E+06	6.19E+06	37	399	100	
	6.18E+06	6.23E+06	398	401	101	

-NL

	AUC T_0	AUC $T_{freeze\text{-}thaw}$	Conc. (µg/L) T_0	Conc. (µg/L) $T_{freeze\text{-}thaw}$	STAB%	AVAREGE STAB%
LLOQ (5 µg/L)	9.10E+04	8.02E+04	4.23	3.58	84.8	91.1
	8.93E+04	9.30E+04	4.13	4.34	105	
	9.22E+04	8.01E+04	4.30	3.58	83.2	
ULOQ (400 µg/L)	6.84E+06	5.91E+06	407	352	86.4	86.9
	6.79E+06	5.91E+06	404	352	87.0	
	6.83E+06	5.95E+06	407	354	87.1	

-PI

	AUC T_0	AUC $T_{freeze\text{-}thaw}$	Conc. (µg/L) T_0	Conc. (µg/L) $T_{freeze\text{-}thaw}$	STAB%	AVAREGE STAB%
LLOQ (1 µg/L)	1.56E+05	1.41E+05	1.01	0.92	90.3	88.9
	1.61E+05	1.40E+05	1.04	0.91	86.9	
	1.60E+05	1.44E+05	1.04	0.93	89.6	
ULOQ (300 µg/L)	4.27E+07	4.07E+07	282	269	95.4	95.5
	4.26E+07	3.99E+07	281	263	93.6	
	4.22E+07	4.11E+07	278	272	97.6	

2.2.8. Figure of average of four calibration curves

-MRM

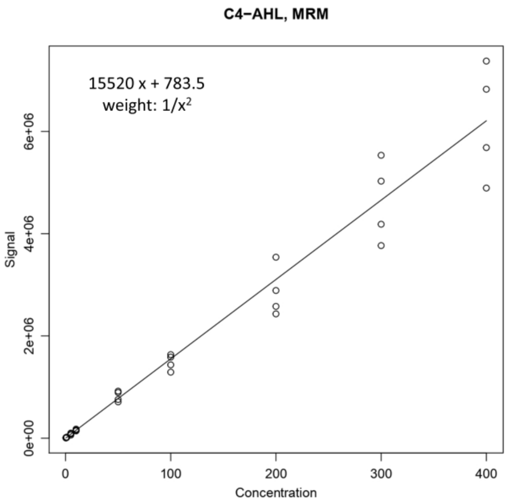

-NL

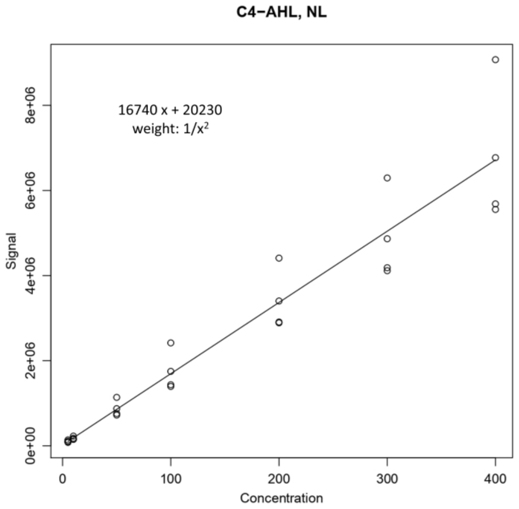

-PI

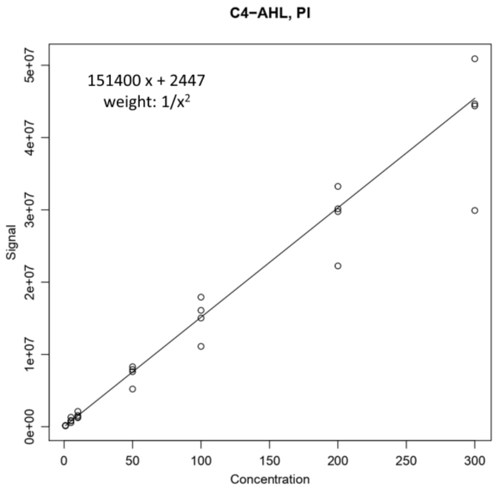

2.3. C7 HQ (2-heptyl-4-hydroxyquinoline)

2.3.1. Calibration curves AUC and equation

-MRM

Conc. (µg/L)	AUC cal. curve 1	AUC cal. curve 2	AUC cal. curve 3	AUC cal. curve 4
0.4	8.02E+03	5.06E+03	5.07E+03	8.51E+03
1	1.83E+04	1.63E+04	1.14E+04	2.31E+04
5	8.53E+04	5.54E+04	6.20E+04	8.01E+04
10	1.99E+05	1.19E+05	1.21E+05	1.46E+05
50	9.01E+05	6.42E+05	6.58E+05	9.74E+05
100	1.81E+06	1.30E+06	1.28E+06	2.02E+06
200	3.72E+06	2.65E+06	2.51E+06	4.06E+06

Linear regressive analysis using a weighting factor of $1/x^2$: y= 15500x + 594.3

-PI

Conc. (µg/L)	AUC cal. curve 1	AUC cal. curve 2	AUC cal. curve 3	AUC cal. curve 4
1	2.56E+05	2.27E+05	2.69E+05	2.30E+05
5	8.24E+05	7.36E+05	6.35E+05	8.15E+05
10	1.69E+06	1.24E+06	1.64E+06	1.47E+06
50	8.32E+06	8.00E+06	7.98E+06	8.12E+06
100	1.43E+07	1.62E+07	1.55E+07	1.43E+07
200	2.95E+07	3.05E+07	3.05E+07	2.90E+07
300	4.47E+07	4.62E+07	4.12E+07	4.00E+07

Linear regressive analysis using a weighting factor of $1/x^2$: y= 146300 x + 96020

2.3.2. Selectivity (SEL%)

- MRM

**Sample**	**AUC**	**Average AUC**	**SEL%**	**16.5**
QS-free_matrix_001	1.01E+03			
QS-free_matrix_002	1.37E+03	1.19E+03		
QS-free_matrix_003	1.19E+03			
STD_400 ppt_001	8.02E+03			
STD_400 ppt_002	5.07E+03	7.20E+04		
STD_400 ppt_003	8.51E+03			

-PI

**Sample**	**AUC**	**Average AUC**	**SEL%**	**4.73**
QS-free_matrix_001	9.14E+03			
QS-free_matrix_002	1.13E+04	1.08E+04		
QS-free_matrix_003	1.20E+04			
STD_1 ppb_001	1.98E+05			
STD_1 ppb_002	2.57E+05	2.29E+05		
STD_1 ppb_003	2.31E+05			

2.3.3. Recovery (REC%)

Sample	AUC	Average AUC
STD_0.4ppb_solv_01	1.52E+04	1.26E+04
STD_0.4ppb_solv_02	1.13E+04	
STD_0.4ppb_solv_03	1.12E+04	
STD_200ppb_solv_01	1.12E+07	1.03E+07
STD_200ppb_solv_02	9.86E+06	
STD_200ppb_solv_03	9.74E+06	
STD_0.4ppb_pls_01	5.98E+03	6.28E+03
STD_0.4ppb_pls_02	6.85E+03	
STD_0.4ppb_pls_03	6.01E+03	
STD_200ppb_pls_01	3.65E+06	3.52E+06
STD_200ppb_pls_02	2.95E+06	
STD_200ppb_pls_03	3.95E+06	
**REC%**	**LLOQ**	**49.9**
	**ULOQ**	**34.3**

2.3.4. Carry-over (CO%)

	Sample	AUC		
MRM	STD_200 ppb	2.59E+06	**CO%**	**16.1**
	QS-free_matrix	1.37E+03		
	STD_400 ppt	8.51E+03		
PI	STD_300 ppb	4.35E+07	**CO%**	**2.98**
	QS-free_matrix	7.04E+03		
	STD_1 ppb	2.36E+05		

2.3.5. Intra-day accuracy (BIAS%) and precision (CV%)

-MRM

Nominal conc. (µg/L)	Real conc. (back-calculated, µg/L)				CV%	BIAS%
	Cal. curve 1	Cal. curve 2	Cal. curve 3	Cal. curve 4		
0.4	0.48	0.29	0.29	0.51	26.5	2.11
1	1.14	1.01	0.70	1.45	25.3	7.09
5	5.46	3.54	3.96	5.13	17.6	10.5
10	12.8	7.61	7.80	9.36	22.3	6.41
50	58.1	41.4	42.4	62.8	18.4	2.27
100	117	83.8	82.6	130	20.1	3.21
200	240	171	162	262	20.7	4.14

-PI

Nominal conc. (µg/L)	Real conc. (back-calculated, µg/L)				CV%	BIAS%
	Cal. curve 1	Cal. curve 2	Cal. curve 3	Cal. curve 4		
1	1.10	0.89	1.19	0.92	11.9	2.28
5	4.97	4.38	3.68	4.91	11.6	11.5
10	10.9	7.80	10.6	9.36	12.6	3.55
50	56.2	54.0	53.9	54.9	1.72	8.68
100	97.1	110	105	97.3	5.34	2.34
200	201	208	208	197	2.19	1.68
300	305	315	281	273	5.84	2.27

2.3.6. LOD and LOQ

-MRM

Sample	AUC		
QS-free_matrix_001	1.43E+03		
QS-free_matrix_002	1.01E+03		
QS-free_matrix_003	9.14E+02		
QS-free_matrix_004	1.13E+03		
QS-free_matrix_005	1.20E+03	Slope	1.55E+04
QS-free_matrix_006	9.03E+02	Intercept	5.94E+02
Average AUC	1.10E+03		
Std. dev QS-free matrix	1.83E+02		**Conc. (µg/L)**
10std. Dev. + Average AUC	2.93E+03	**LOQ**	**0.151**

-PI

Sample	AUC		
QS-free_matrix_001	1.20E+04		
QS-free_matrix_002	7.04E+03		
QS-free_matrix_003	1.37E+04		
QS-free_matrix_004	1.51E+04		
QS-free_matrix_005	1.46E+04	Slope	1.46E+05
QS-free_matrix_006	1.38E+04	Intercept	9.60E+04
Average AUC	1.27E+04		
Std. dev QS-free matrix	2.71E+03		**Conc. (µg/L)**
10std. Dev. + Average AUC	3.98E+04	**LOQ**	**0.385**

2.3.7. Freeze-thaw stability (STAB%)

-MRM

	AUC T_0	AUC $T_{freeze\text{-}thaw}$	Conc. (µg/L) T_0	Conc. (µg/L) $T_{freeze\text{-}thaw}$	STAB%	AVAREGE STAB%
LLOQ (0.4 µg/L)	8.02E+03	8.51E+03	0.48	0.51	107	115
	5.07E+03	6.67E+03	0.29	0.39	136	
	7.06E+03	7.25E+03	0.42	0.43	103	
ULOQ (200 µg/L)	3.72E+06	4.06E+06	240	262	109	118
	2.51E+06	3.23E+06	161	209	129	
	2.65E+06	3.04E+06	171	196	115	

-PI

	AUC T_0	AUC $T_{freeze\text{-}thaw}$	Conc. (µg/L) T_0	Conc. (µg/L) $T_{freeze\text{-}thaw}$	STAB%	AVAREGE STAB%
LLOQ (1 µg/L)	2.98E+05	2.65E+05	1.38	1.16	83.7	90.2
	2.12E+05	1.99E+05	0.79	0.70	88.8	
	3.01E+05	2.97E+05	1.40	1.37	98.1	
ULOQ (300 µg/L)	4.23E+07	3.99E+07	289	272	94.3	96.2
	4.98E+07	4.59E+07	340	313	92.1	
	4.03E+07	4.12E+07	275	281	102	

2.3.8. Figure of average of four calibration curves

-MRM

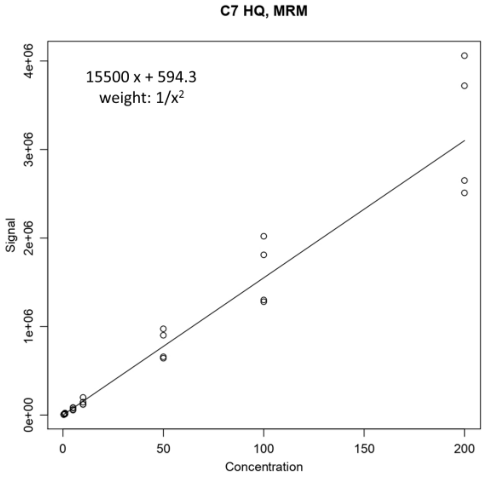

-PI

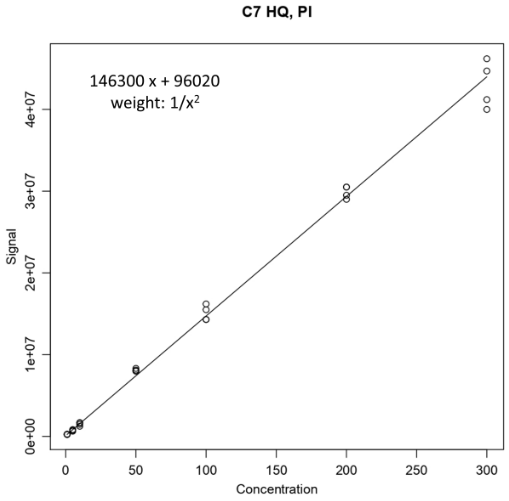

3. Tables

Table S1 Multiple Reaction Monitoring (MRM) scan parameters and selected transition for AHLs and HQs analysis (bolded transitions were used as quantitative ones). DP: Declustering Potential; EP: Entrance Potential; CE: Collision Energy; CXP: Collision Cell Exit Potential

Analyte	Precursor ion $[M+H]^+$	Product Ion $[M+H]^+$	DP (Volts)	EP (Volts)	CE (Volts)	CXP (Volts)
3-oxo-C12-AHL	298.2	102.2	109	10.0	26.9	12.0
	298.2	197.2	109	10	20.9	20
C4-AHL	172.1	102.2	49.0	10.0	12.0	13.0
	172.1	71.1	49.0	10.0	12.0	13.0
C7 HQ	260.0	188.0	290.0	14.0	42.1	10.0
	260.0	147.0	290.0	14.0	49.2	13.0
ND3	203.2	102.1	65.0	6.0	23.0	6.0
	203.2	74.1	65.0	6.0	20.0	7.0

Table S2 Neutral Loss (NL) and Product Ion (PI) scan methods parameters for AHL and HQ signalling molecules analysis. DP: Declustering potential; EP: Entrance Potential; CE: Collision Energy; CXP: Collision Cell Exit potential

Molecules family	MS Mode	Δm (Da)	DP (Volts)	EP (Volts)	CE start (Volts)	CE stop (Volts)	CXP start (Volts)	CXP stop (Volts)
AHL	NL	101.0	109.0	10.0	15.0	25.0	9.0	13.0
	PI	102.0	110.0	9.0	15.0	25.0	9.0	12.0
HQ	PI	175.0	110.0	9.0	35.0	45.0	9.0	11.0

Table S3 Chemical formula, *m/z* ratio ($[M+H]^+$) and proposed structural protonated formula of detected AHL compounds with untargeted approach

N° of carbon atoms	Compound	Chemical formula	$[M+H]^+$	Proposed chemical protonated structure
6	C_6-AHL	$C_{10}H_{17}NO_3$	200.1287	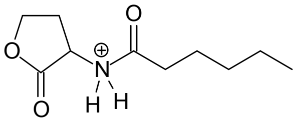
10	3-oxo-C_{10}-AHL	$C_{14}H_{23}NO_4$;	270.1705	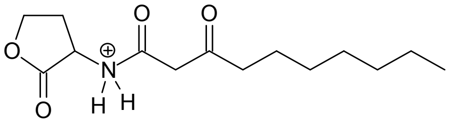
12	C_{12}-AHL	$C_{16}H_{29}NO_3$;	284.2226	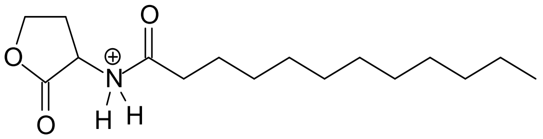

Table S4 Chemical formula, *m/z* ratio ($[M+H]^+$) and proposed structural protonated formula of detected HQ compounds with untargeted approach. The structure of the species with an unsaturation on the acyl-chain (such as C6:1-HQ), due to the uncertainty of the position of the double bond along the chain, are not reported

N° of carbon atoms	Compound	Chemical formula	$[M+H]^+$	Proposed chemical structure
2	C_2-HQ	$C_{11}H_{11}NO_2$	190.0868	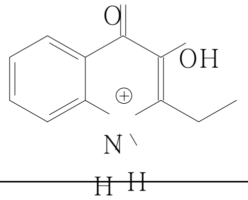
3	C_3-HQ	$C_{12}H_{13}NO_2$	204.1024	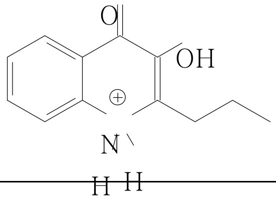
4	C_4-HQ	$C_{13}H_{15}NO_2$	218.1181	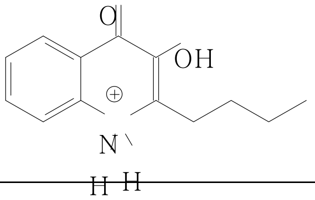

5	C_5-HQ	$C_{14}H_{17}NO_2$	232.1337	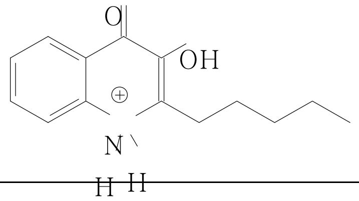
6	C_6-HQ	$C_{15}H_{19}NO_2$	246.1494	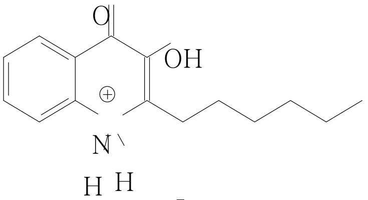
	$C_{6:1}$-HQ	$C_{15}H_{17}NO_2$	244.1337	-
7	C_7-HQ	$C_{16}H_{21}NO_2$	260.1650	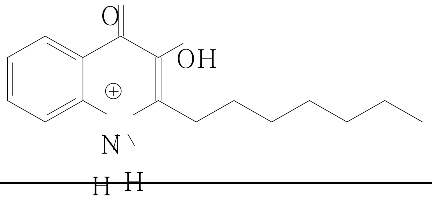
8	C_8-HQ	$C_{17}H_{23}NO_2$	274.1807	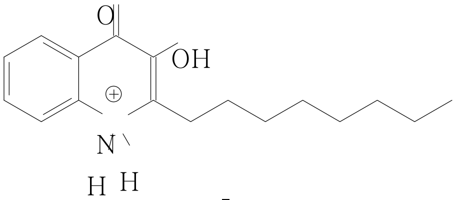
	$C_{8:1}$-HQ	$C_{17}H_{21}NO_2$	272.1650	-
9	C_9-HQ	$C_{18}H_{25}NO_2$	288.1936	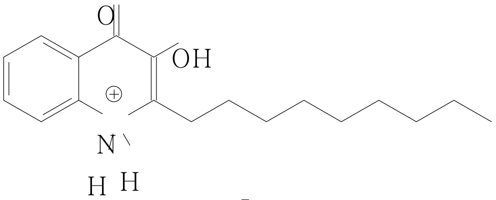
	$C_{9:1}$-HQ	$C_{18}H_{23}NO_2$	286.1807	-
11	C_{11}-HQ	$C_{20}H_{29}NO_2$	316.2276	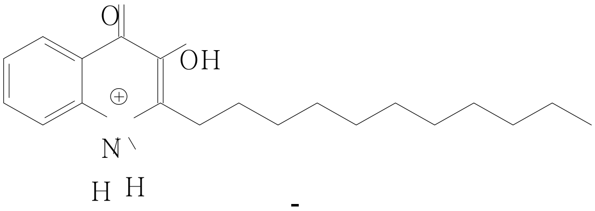
	$C_{11:1}$-HQ	$C_{20}H_{27}NO_2$	314.2120	-

Table S5 Results obtained from the study of the calibration models. The p-values obtained for the study of heteroscedasticity (F-test and Levene test), of the quadraticity (Partial F-test), and of the standardized residuals were considered significant if lower than 0.05 and reported in bold in the Table. The weights and the equations of the calibration models were obtained using an R routine

		Study of heteroscedasticity					
Analyte	Calibration range (µg/L)	F-test (p-value)[a]	Levene test (p-value)[a]	Weight	Partial F-test for quadraticity (p-value)[a]	Equation of the calibration curve	Normality of standardized residuals (p-value)[a]
3-oxo-C12-AHL, MRM	0.4–400	**1.2×10^{-9}**	**3.0×10^{-2}**	$1/x^2$	0.73	141900 x – 2609	0.99
3-oxo-C12-AHL, NL	1 – 300	**1.4×10^{-8}**	**2.5×10^{-2}**	$1/x^2$	0.68	490900 x + 152400	0.98
3-oxo-C12-AHL, PI	5 – 400	**7.3×10^{-8}**	**8.7×10^{-4}**	$1/x^2$	0.34	709400 x + 93520	0.71
C4-AHL, MRM	0.4 – 400	**2.7×10^{-9}**	**4.3×10^{-7}**	$1/x^2$	0.52	15520 x + 783.5	0.83
C4-AHL, NL	5 – 400	**6.0×10^{-6}**	7.2×10^{-1}	$1/x^2$	0.96	16740 x + 20230	0.99
C4-AHL, PI	1 – 300	**3.0×10^{-8}**	1.9×10^{-1}	$1/x^2$	0.28	151400 x + 2447	0.95
C7 HQ, MRM	0.4 – 200	**2.4×10^{-8}**	**8.5×10^{-11}**	$1/x^2$	0.53	15500 x + 594.3	0.29
C7 HQ, PI	1 – 300	**5.8×10^{-7}**	**1.7×10^{-8}**	$1/x^2$	0.80	146300 x + 96020	0.99

[a] 95% level of significance (p-value < 0.05)

Table S6 Back calculation results

	Deviation (%)								
Analyte ↓| Concentration (ppb) →	**0.4**	**1**	**5**	**10**	**50**	**100**	**200**	**300**	**400**
**3-oxo-C12-AHL, MRM**	2	-5	0	2	11	8	0	-1	-2
**3-oxo-C12-AHL, NL**	\	\	4	-9	8	-5	1	-4	4
**3-oxo-C12-AHL, PI**	\	2	-10	-6	3	3	3	5	\
**C4-AHL, MRM**	-1	0	7	1	6	-4	-8	-1	0
**C4-AHL, NL**	\	\	3	-6	2	3	1	-4	1
**C4-AHL, PI**	\	-3	17	3	-4	-1	-5	-7	\
**C7 HQ, MRM**	-2	8	-10	-6	2	3	4	\	\
**C7 HQ, PI**	\	2	-10	-3	10	2	2	-2	\
